# Definition, Prevalence and Management of Dyslipidemia in Patients and Survivors of Childhood and Adolescent Cancer—A Systematic Review

**DOI:** 10.3390/cancers18050837

**Published:** 2026-03-04

**Authors:** Fiona L. Wagenseil, Luca Bühlmann, Stephanie B. Dixon, Matthew J. Ehrhardt, Sarah P. Schladerer, Cornelia Vetter, Maria Otth, Katrin Scheinemann

**Affiliations:** 1 Faculty of Health Sciences and Medicine, University of Lucerne, 6002 Lucerne, Switzerland; 2 Department of Epidemiology and Cancer Control, St. Jude Children’s Research Hospital, Memphis, TN 38105-3678, USA; 3 Department of Oncology, St. Jude Children’s Research Hospital, Memphis, TN 38105-3678, USA; 4 Division of Oncology-Hematology, Children’s Hospital of Eastern Switzerland, 9006 St. Gallen, Switzerland; 5 Department of Oncology, University Children’s Hospital Zurich-Eleonore Foundation, 8008 Zurich, Switzerland

**Keywords:** dyslipidemia, cholesterol, HDL-cholesterol, LDL-cholesterol, triglycerides, pediatric cancer, cancer survivors, cancer management

## Abstract

**Background/Objectives:** There is little information on the definition and management of dyslipidemia in patients and survivors of childhood, adolescent and young adult (CAYA) cancer. However, it is known that this population is at higher risk of developing cardiovascular disease (CVD). Dyslipidemia, hypertension, and metabolic syndrome are common among CAYA cancer survivors due to the cancer itself or the treatment received. Therefore, managing dyslipidemia in this population is crucial to mitigate the risk of long-term CVD. The aim of this systematic review was to summarize currently used definitions and cutoffs for dyslipidemia, its prevalence, and management strategies in CAYA cancer survivors. This review further describes reported pharmacological and lifestyle interventions and their impact on lipid levels. **Methods**: We conducted a systematic literature search in PubMed, including studies published in English, German or French between January 2015 and February 2025. The population included individuals diagnosed with any type of CAYA cancer prior to 25 years of age and either receiving cancer treatment or in follow-up care. We considered all types of publications except for Phase I and II studies. We followed PRISMA guidelines, assessed the quality of the eligible studies according to the respective Joanna Briggs Institute’s Critical Appraisal Tools, and reported the results descriptively. **Results**: Of 575 identified publications, 53 fulfilled the inclusion criteria. Forty-three studies reported on the definitions of abnormal lipid values, 40 stated the prevalence of abnormal lipid values, and 17 studies described management approaches, of which 12 were case reports and small case series. For all three outcomes, the results were very heterogeneous. Using the example of triglycerides (TGs), the cutoff values for high TGs ranged from 5.17 mmol/L to 6.2 mmol/L and the reported prevalence of high TGs ranged from 0% to 75%, with an average of 31%. The only reported intervention to prevent dyslipidemia in CAYA cancer survivors was lifestyle modification. Preventive strategies that started during treatment were lifestyle modifications and fish oil supplements. **Conclusions:** Our systematic review provides a comprehensive overview of existing definitions, prevalences, and management of abnormal lipid values in CAYA cancer patients and survivors. However, the identified heterogeneities indicate that reported prevalences and results of interventions must be interpreted cautiously. An internationally harmonized approach to defining and reporting lipid values in CAYA cancer survivors is urgently needed to enable tailored screening and treatment strategies.

## Simple Summary

Dyslipidemia is a relevant risk factor for premature cardiovascular disease, which contributes to long-term complications such as atherosclerosis, heart attack, or stroke. We know that patients and survivors of childhood cancer have an increased risk of cardiovascular disease due to the oncological treatment they have received. However, the definition of dyslipidemia is very heterogeneous in this population, which influences reporting of the prevalence of abnormal lipid values and its management. In this systematic review, we included 53 studies to provide an overview of the currently used definitions and cutoffs for dyslipidemia or abnormal lipid values and its prevalence in patients and survivors of childhood and adolescent cancer. This review further describes different approaches to treat and prevent dyslipidemia in this population.

## 1. Introduction

Advances in cancer therapies, risk stratifications, and supportive care have resulted in an increase in 5-year survival in childhood, adolescent and young adult (CAYA) cancer survivors from 5–30% in the early seventies to now around 85% worldwide [1,2]. Nevertheless, CAYA cancer survivors experience late effects from the cancer itself and its treatment, leading to an increased burden in long-term chronic health conditions (i.e., late effects). Late effects can potentially affect every organ system. In addition to morbidity, mortality due to cardiovascular disease (CVD) is higher in CAYA cancer survivors compared to the general population [3,4,5,6,7]. Knowing that CAYA cancer survivors experience higher morbidity and mortality, risk-stratified long-term follow-up care to mitigate the development and/or progression of late effects is of high importance. Different national and international guidelines exist to perform this risk-adapted screening. Cardiovascular health is part of all these guidelines, whereas screening for dyslipidemia is not covered in all guidelines [8,9,10].

The term dyslipidemia refers to a metabolic disorder with changes in lipid metabolism and lipoprotein transport. These changes are primarily manifested by abnormal low-density lipoprotein cholesterol (LDL-C), high-density lipoprotein cholesterol (HDL-C), total cholesterol (TC) and triglyceride (TG) levels. Different definitions of dyslipidemia are used today, such as at least one lipid parameter above or below the normal range or treatment with lipid-lowering medications [11,12,13]. Among the general population, hypercholesterolemia is the most frequent type of dyslipidemia, and high LDL-C is a major risk factor for CVD [14]. In recent decades, the global prevalence of dyslipidemia has significantly increased due to epidemiological and demographic growth and unhealthy changes in nutrition and lifestyle. In the United States alone, the prevalence of dyslipidemia has increased from 17% to 38% from 2015 to 2018 [15]. According to a recent meta-analysis, the global prevalence of hypertriglyceridemia is 29%, 24% for hypercholesterolemia, 38% for low HDL-C, and 19% for high LDL-C [16]. The use of lipid-lowering agents has substantially lowered cholesterol levels and subsequent cardiovascular mortality in high-income countries [14]. Besides treatment with lipid-lowering agents, a healthy lifestyle, including attention to both diet and physical activity, is strongly recommended to reduce the risk of CVD [17,18].

Cardiovascular disease contributes to a relevant proportion of late morbidity and mortality in CAYA cancer survivors. A study by Bhakta et al. examined chronic health conditions, including dyslipidemia, ranging from asymptomatic to fatal. The cumulative incidence for any cardiovascular chronic health condition at the age of 50 years was 93.2% (95% CI 92.4–94.0) [19]. Besides dyslipidemia, CAYA cancer survivors are at a higher risk for other components of metabolic syndrome, including insulin resistance, obesity, and hypertension, which occurs more frequently in CAYA cancer survivors compared to persons without a history of cancer [20].

To reduce morbidity and mortality due to dyslipidemia in the general population, international guidelines such as the American College of Cardiology/American Heart Association (ACC/AHA) or the European Society of Cardiology (ESC) emphasize the importance of early lipid control [18,21]. Despite the high burden of CVD in CAYA cancer survivors, evidence regarding the definition, prevalence, and management of dyslipidemia in this population remains limited and inconsistent across existing studies [22]. Measuring lipid parameters in CAYA cancer survivors might allow for specifically targeted management of dyslipidemia through either lifestyle modifications or the implementation of lipid-lowering therapy.

The aim of this systematic review was to summarize the current evidence on the definition, prevalence, and management of dyslipidemia in patients and survivors of CAYA cancer.

## 2. Materials and Methods

This systematic review was conducted and reported in accordance with the Preferred Reporting Items for Systematic Reviews and Meta-Analyses (PRISMA) 2020 guidelines (https://www.prisma-statement.org/). The protocol was developed using the PICO framework (Population, Intervention, Comparison, and Outcomes) and focused on CAYA cancer patients and survivors in whom dyslipidemia was assessed, monitored, or managed.

The literature search was performed through PubMed on 23 February 2025 using four concepts: (1) cancer diagnoses, (2) CAYA population, (3) dyslipidemia, and (4) treatment/management. Each concept consisted of several key words and MeSH terms (mh, Supplementary Table S1). The considered publication period ranged from 1 January 2015 to 1 February 2025. All types of publications were included except for Phase I and II studies. Language was restricted to English, French and German. We used Rayyan (https://www.rayyan.ai) for the Title and Abstract (TiAb) screening. Each TiAb was screened for eligibility by two independently working researchers, followed by a full-text review of potentially eligible studies. Discrepancies during TiAb and full-text screening were resolved through discussion or consultation with a third reviewer. For reviews covering the outcome of interest, we screened the references. For additionally identified titles, we performed additional TiAb and full-text screening.

Publications were eligible if they examined dyslipidemia in CAYA diagnosed with cancer. CAYA were defined as participants ≤25 years of age at cancer diagnosis or if the average age was ≤20 years when no range was provided. All cancer types were considered, and both patients undergoing treatment and survivors who had completed treatment were eligible. Publications were required to report data related to dyslipidemia, either explicitly using the term “dyslipidemia” or by reporting specific lipid parameters, including total cholesterol, LDL-C, HDL-C, or TGs. Publications were excluded if they reported metabolic syndrome as a combined outcome without providing specific data on lipid parameters.

We extracted the characteristics of each eligible publication in predefined data extraction sheets. Each data extraction sheet was verified by a second reviewer. The data extraction sheet included information on author, publication year, study type, treatment era, number and age of survivors, diagnosis, the timepoint of lipid assessment, the definition of abnormal lipid values/dyslipidemia, the prevalence of abnormal lipid values, and the results of interventions, if applicable. Some studies reported cutoff values for lipid values in mg/dL and others in mmol/L. We converted all values into mmol/L and mg/dL using the omni-calculator (https://www.omnicalculator.com/health/cholesterol-units (accessed on 15 August 2025). If publications reported lipid values as “borderline” and “high”, we only considered the “high” values in the manuscript but show all results in the Supplementary Material (Supplementary Table S2).

To summarize the prevalence of abnormal lipid values, we made the following three methodological decisions: (1) For reporting on prevalence, we excluded case reports and small case series as they are not conclusive for the purpose of this review. (2) If studies reported abnormal lipid values of different severities (e.g., moderately and highly increased values), we summed them up into one category. (3) If a study reported results from an intervention and control group where both groups were survivors, we only stated the prevalence of the control group. For clearer presentation we grouped the prevalences into categories of 20%. The original values are shown in the Supplementary Material (Supplementary Table S4). Finally, we calculated the mean of the reported prevalences per category.

We used the critical appraisal tools from the Joanna Briggs Institute (JBI) (https://jbi.global/critical-appraisal-tools (accessed on 27 August 2025) to assess the included publications’ quality and risk of bias. We applied the critical appraisal checklists for randomized controlled trials (RCTs), cross-sectional studies, cohort studies, case control studies, case reports, and studies reporting prevalence. Each checklist consists of eight to thirteen criteria depending on the type of publication. As the JBI does not provide a rating scale for publication quality, we defined three quality categories. Criteria judged as “not applicable” were not considered in the quality assessment. We defined “Quality 1” if publications met all criteria, “Quality 2” if publications did not meet one or two criteria of the respective checklist, and “Quality 3” if publications did not meet three or more criteria. Based on the anticipated heterogeneity, results are presented descriptively only. Each critical appraisal checklist was completed by one reviewer and checked by a second one. This review is not registered.

## 3. Results

### 3.1. Description of Included Studies

**Figure 1:**
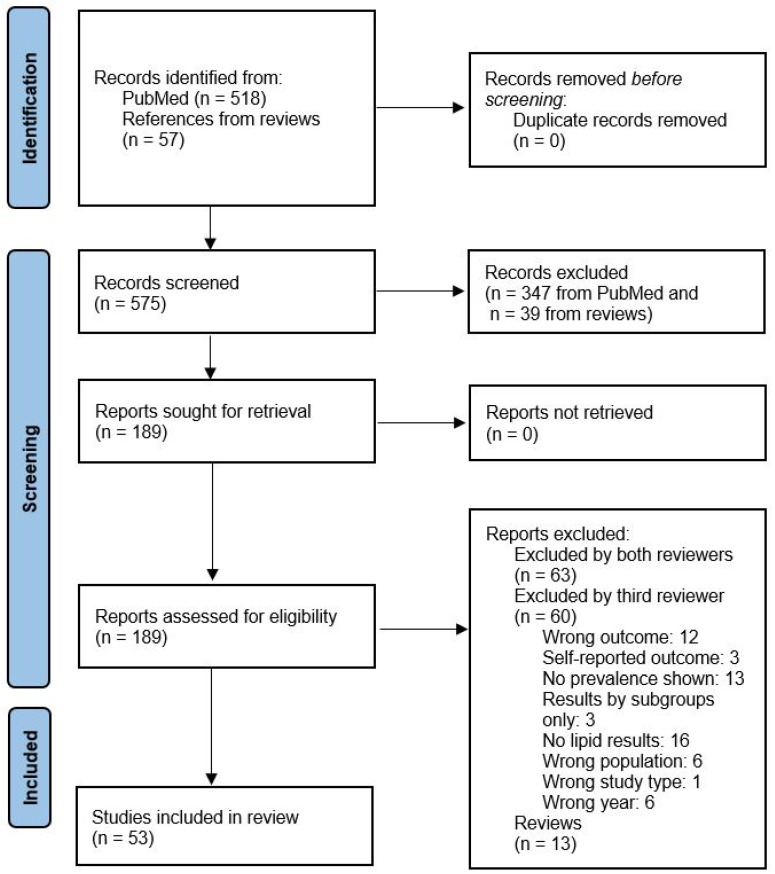
PRISMA 2020 flow diagram for the identification of included publications (https://www.prisma-statement.org/).

We identified 575 publications in PubMed and additionally included 57 references from systematic reviews. Following the TiAb screening, 189 publications remained for the full-text screening. Ultimately, 53 publications were selected, critically evaluated, and summarized for this systematic review (Figure 1). The characteristics of the 53 included studies are provided in Table 1.

Most of the included publications were categorized as Quality 2 (Table 1). The most frequent reasons for down-grading were no identification of confounding factors and no strategies to deal with them (Supplementary Table S6).

### 3.2. Definition of Dyslipidemia by Lipid Parameter

Table 2 provides an overview of the definitions reported for dyslipidemia, sorted by lipid parameter. Despite the heterogeneity of cutoffs used, we could identify a definition for each lipid parameter that was used most frequently.

#### 3.2.1. Triglycerides

In most of the included publications, the cutoff for high TGs for adults was set at ≥1.7 mmol/L [13,20,22,23,25,26,27,28,30,31,32,33,34,35,36,38,42,45,46,54,55,57,58,59,61,62,64,65]. Higher values (>2.26 mmol/L and >3.39 mmol/L) were used in four studies [37,41,43,67], and a lower value was used in one study [55]. Nine publications used separate cutoffs for children and adolescents [11,13,25,27,28,43,44,54,60]. Six publications even took different age limits for children into account [25,27,28,44,54,60], where an age of 9 and 10 years were used in five studies. A value ≥1.46/1.47 mmol/L was mentioned in seven publications as high in children [13,25,27,28,43,44,54] (Table 2).

#### 3.2.2. LDL-C

The cutoff for high LDL-C was set at >3.36/3.4 mmol/L in nine publications and was used in adults and children [13,26,28,37,38,44,45,54,59] (Table 2). Four publications set higher values [22,27,41,67] and two set lower values in children [55,62].

#### 3.2.3. HDL-C

Fourteen publications reported gender-specific cutoffs for HDL-C, with <1.03 mmol/L in men and <1.3 mmol/L in women [13,20,22,23,28,32,34,38,45,51,54,57,58,61,62,64,65,69]. For the same number of publications, the cutoff was stated at <1.03 mmol/L, regardless of the gender [21,22,28,30,35,41,42,54,55,57,58,61,62,65]. Two publications reported separate cutoffs for children depending on their age [11,31] (Table 2).

#### 3.2.4. Total Cholesterol

High total cholesterol defined as ≥5.17 mmol/L was most frequently mentioned [24,26,33,37,41,44,45,54,59]. Two publications used lower cutoffs [62,67] and five used higher cutoffs [27,42,43,46,55] (Table 2).

### 3.3. Prevalence of Dyslipidemia

**Figure 2:**
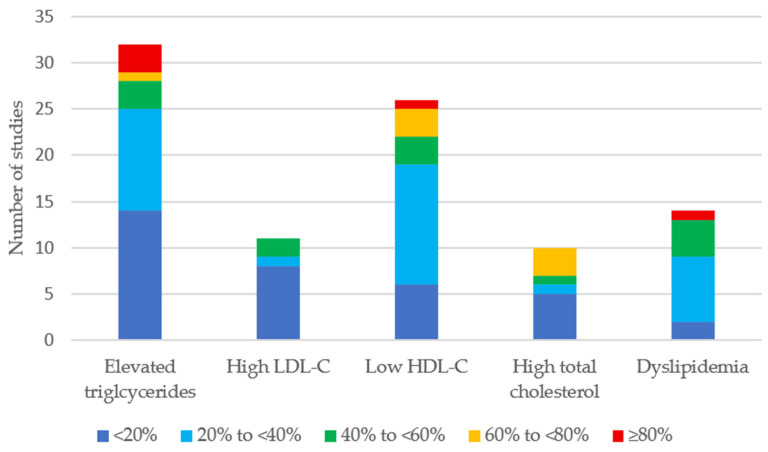
Distribution of the prevalence of dyslipidemia among the included publications.

The reported prevalence of abnormal lipid parameters and dyslipidemia (as defined in the respective publications) was very broad (Table 3, Figure 2). Elevated TGs and low HDL-C were the parameters most frequently reported as abnormal (Figure 2). Among all five parameters assessed, the prevalence remained below 40% in most publications. A proportion of ≥80% abnormal values was reported in three different publications for high TGs [24,30,44] and in one for low HDL-C and dyslipidemia. This study assessed dyslipidemia at diagnosis of leukemia [11]. Elevated LDL-C was reported in eleven studies, with no study reporting a prevalence higher than 47.6% [11,13,22,27,30,36,37,44,50,54,59]. High total cholesterol was reported in ten studies [11,24,27,29,30,31,37,41,44,59].

### 3.4. Prevention and Management of Dyslipidemia

Five publications reported interventions to improve abnormal lipid parameters or to prevent its development [24,37,44,49,56] (Table 4). Two studies analyzed the effect of Omega-3 fatty acid supplementations [24,49]. Three studies aimed to improve high lipid parameters by promoting a healthy lifestyle, including physical, psychological support, and healthy eating recommendations [37,44,56]. The health behavior and lifestyle intervention used by Delorme et al. (healthy eating habits, individualized and guided training programs, and psychological support) showed a trend towards a lower proportion of survivors with high LDL-C in the intervention group after 15 months [37]. Javalkar et al. used a combination of lifestyle modifications and started pharmacotherapy if guideline-defined thresholds were not met after 6 months. The change in median lipid parameters showed a trend towards larger improvement in the intervention group compared to controls [44]. Napartuk et al. analyzed a nutritional intervention where after one year, a trend towards an increase in HDL-C and a decrease in LDL-C could be observed [56]. One study additionally started lipid-lowering agents (name not mentioned) if lifestyle modifications did not show the anticipated effect [63].

The results from case reports and small case series are summarized in the Supplementary Table S5. All these publications report the management of dyslipidemia at cancer diagnosis or during cancer treatment. The approaches were individualized and determined by the level of lipid values measured. Therefore, the results cannot be generalized. The reported management ranged from a “spontaneous” decrease of lipid levels in leukemia patients once the oncological treatment was initiated to lipid-lowering agents use or, in extreme cases, lipid apheresis [33,40,42,46,48,52,55,63,71].

## 4. Discussion

This systematic review summarized the literature from the last 10 years regarding the definition, prevalence, and management of dyslipidemia in CAYA cancer patients and survivors.

The definition of dyslipidemia and the cutoffs for abnormal lipid parameters were very heterogeneous in the included studies. Still, for TGs, many included studies set the cutoff at ≥1.7 mmol/L (≥150 mg/dL) [13,20,22,23,25,26,27,28,30,31,32,33,34,35,36,38,42,45,46,54,55,57,58,59,61,62,64,65] and most LDL-C cutoffs were mentioned at ≥3.36/3.4 mmol/L (≥130/132 mg/dL) [13,26,28,37,38,44,45,54,59]. The heterogeneity in cutoffs used was true for the pediatric population but also for adults. This is not surprising, as different national and international recommendations exist in adults [14]. The recommendations include cutoff values to define abnormal values and treatment goals for patients receiving lipid-lowering agents. Recommendations are provided by the European Society of Cardiology together with the European Atherosclerosis Society, the “German Society of Lipology” (Deutsche Gesellschaft für Lipidologie) based on the SCORE tool, or the American Association of Clinical Endocrinology. Taking the example of LDL-C, the German Society of Lipology uses risk-stratified cutoff values. The cutoff is set at <3 mmol/L (<116 mg/dL) for patients with a low risk and decreases to <1.4 mmol/L (<55 mg/dL) for very high-risk situations [74]. The value of <1.4 mmol/L (<55 mg/dL) corresponds to the one stated by “The Task Force for the management of dyslipidemias of the European Society of Cardiology (ESC) and European Atherosclerosis Society (EAS)” for primary prevention in patients with heterozygous familial hypercholesterolemia [21]. The cutoff values stated by the “American Association of Clinical Endocrinology” are slightly less strict, with <3.4 mmol/L (<130 mg/dL) in the general population or <1.8 mmol/L (<70 mg/dL) in people with atherosclerotic CVD [75]. Besides LDL-C, many recommendations target triglycerides and use risk-stratified approaches to estimate the patients’ individual risk and to start lipid-lowering treatment.

In addition to recommendations, many tools exist to assess the cardiovascular risk in the general population: Framingham [76], HFA-ICOS (Heart Failure Association of the European Society of Cardiology Cardio-Oncology Study Group in collaboration with the International Cardio-Oncology Society) [77], SCORE2 (Systemic Coronary Risk Estimation) [78], ASSIGN [79], QRSIK3 [80] and PROCAM (Prospective Cardiovascular Munster Study) [81]. The SCORE2 and Framingham risk assessments are the most frequently used ones [82,83].

Until now, no consideration has been given as to how factors uniquely associated with dyslipidemia among CAYA cancer patients and survivors (e.g., cancer treatment exposures) may influence risk-stratification. However, these considerations are crucial and would influence whether CAYA cancer patients have the same cutoffs for starting treatment as the general population or whether lower values must be applied.

As shown in Figure 2, high TGs and low HDL-C are the most common abnormal lipid parameters in CAYA cancer patients and survivors. These results are similar to patterns in the general population. Ballena-Caicedo et al. calculated the global prevalence of dyslipidemia and abnormal lipid values in the general population [16]. The global prevalence of high total cholesterol and high LDL-C is 24.09% and 18.93%, respectively. The average prevalence of these parameters in our included studies was 31% and 18%. As we observed, high TGs and low LDL-C were most prevalent. The global prevalence in the publication by Ballena-Caicedo et al. for high TGs was 28.7% and 28.43% for low HDL-C. These proportions are similar to the average prevalence in the CAYA population of the current systematic review, with 29% for high TG sand 35% for low HDL-C. However, it is important to consider that the range of abnormal lipid values was very broad in the studies included in our review. Although the overall prevalence appears to be similar, it is significantly higher in subgroups of CAYA cancer survivors [20], and the prevalence in some studies is probably also influenced by the rather small sample sizes [24,31,33,35,36,37,40,42,44,46,47,48,49,52,55,56,59,62,67,68,69,70,71]. All nine studies that reported on abnormal lipid values at cancer diagnosis or during treatment were case reports or small case series (Supplementary Table S5) and were therefore not included in the analysis of this systematic review. Depending on the diagnosis and treatment, abnormal lipid values are frequent during treatment and resolve spontaneously. In addition, these transient abnormal lipid values most probably do not contribute to the long-term cardiovascular morbidity in survivors, in contrast to abnormal lipid values during survivorship care.

Five publications mentioned interventions to prevent the development of dyslipidemia [24,37,44,49,56]. In both studies that assessed the benefit of Omega-3 supplementation during leukemia treatment, TGs decreased over time [24,49]. Laumann et al. additionally assessed the impact on total cholesterol, which decreased as well [49]. In a meta-analysis of 90 randomized controlled trials, Wang et al. investigated the association of Omega-3-fatty acid intake and dyslipidemia [17]. They found evidence for a dose-response relationship with a nearly linear decrease in TGs and non-HDL-C under the intake of Omega-3 fatty acids. They further postulate that patients with a higher risk for cardiovascular disease (e.g., overweight, obesity, hyperlipidemia) may experience a larger effect [17]. The other three publications assessed the impact of different forms of lifestyle modifications on the lipid parameters over time [37,44,56]. Due to the different methodological approaches, the results of these three publications are not directly comparable. However, the studies show trends towards improvement in total cholesterol [44,56], HDL-C [56], LDL-C [37,56], and TGs [44]. The study by Javalkar et al. showed a larger positive trend in changes for HDL-C and LDL-C in controls over time. None of the studies showed a significant improvement in abnormal lipid values following an intervention. This is surprising as the positive effect of lifestyle modifications is well known and implemented in many guidelines to prevent CVD [16,18,21]. Some studies also show a positive impact of specific lifestyle changes on lipid levels in adults. A reduction of excessive body weight can improve triglyceride, LDL-C and HDL-C levels [84,85]. However, only five publications were eligible to assess the impact of interventions in the CAYA cancer population. Their methodological approaches, as well as the study populations, were heterogeneous, which influences the results. In addition, all except one study were performed during cancer treatment, where additional risk factors for dyslipidemia exist (e.g., less physically active and receiving treatment with Asparaginase or steroids).

There are two clinical implications resulting from this review. First, dyslipidemia is quite frequent in CAYA cancer survivors. Clinicians should be aware of it and screening should be performed on a regular basis. However, our results do not provide sufficient information to formulate recommendations. In addition, current long-term follow-up care guidelines, where dyslipidemia is mentioned among many other outcomes, are inconsistent regarding the time point and frequency of screening [8,86,87]. A harmonized recommendation from the International Guideline Harmonization Group (IGHG) specifically for dyslipidemia is currently under development (www.ighg.org). In the IGHG guideline on metabolic syndrome, dyslipidemia was part of the composite outcome and no specific recommendations were formulated [88]. Second, we recommend using official cutoff values and not local ones for lipid values to allow comparison in any publication.

Our methodologically rigorous approach is a strength of this systematic review, including the TiAb and full-text screening completed by two reviewers independently, and discordances were reviewed by a third independent researcher. Also, we followed the strict methodological approach of PRISMA guidelines for systematic reviews. The limitations of this review are linked to the information provided in the included studies. Due to the heterogeneity in the CAYA cancer patient and survivor cohorts, generalizability is difficult, and the average prevalence of abnormal lipid values must be interpreted cautiously. This is because some studies include all diagnoses, others included only leukemia survivors without HSCT and others included transplanted survivors only. In addition, the small sample size is a limitation in some studies. We tried to overcome this limitation by excluding case reports and small case series from some analyses.

## 5. Conclusions

Our systematic review demonstrates that dyslipidemia is prevalent in a relevant proportion of CAYA cancer patients and survivors. Due to the heterogeneity in the populations described and the cutoffs used to define abnormal lipid parameters, it is not possible to draw a general conclusion regarding the exact prevalence and suggested management, identifying a major gap among existing studies and highlighting two key topics for future research. First, a consensus on uniform definitions for abnormal lipid values is needed. Second, we must explore whether the same cutoff values can be used for CAYA cancer survivors as for the general population or whether the cutoffs to start an intervention should be set at lower levels.

## Figures and Tables

**Table 1:** Overview of the included studies.

First Author, Publication Year	Study Type. Outcome Type *	Treatment Era. Number of Survivors (=N)	Diagnosis	Age at Diagnosis [Years]° Follow-Up [Years]°	Timepoint of Lipid Assessment	Quality Assessment
Armstrong, 2015 [23]	Prospective cohort (SJLIFE). D, P	Diagnosed 1962–2012. N = 3029	ALL	0–4 (33.8%), 5–9 (23.7%), 10–14 (24.1%), 15–19 (17.5%), >10 (0.9%) Follow-Up since Dx: 22.6 (10.4–48.3)	Once during follow-up in 10-year survivors	2
Barbosa-Cortes, 2023 [24]	Prospective RCT (Phase 2). D, T	NR, from clinicaltrials.gov: study start 09/2010–08/2016 last patient examined. N = 34	ALL	Mean ± SD: 6.7 ± 2.7 Follow-Up (not defined): 3 months	During treatment: baseline and 3 months	2
Barbosa-Cortes, 2017 [25]	Prospective cohort. D, P	NR. N = 52	Leukemia, lymphoma	12.1 (7.1–17) Follow-Up since EOT: 4 (2–5)	During follow-up: baseline and 6 months	2
Bayram, 2017 [26]	Prospective cross-sectional. D, P	Diagnosed 01/2003–02/2009. N = 60	ALL	5 (1.7–13) Follow-Up since EOT: 4 (2–10.1)	Once during follow-up	2
Bélanger, 2023 [27]	Prospective case control and cohort. D, P	Recruited 06/2017–10/2019. N = 50	All types of childhood cancer	Age at evaluation: mean ± SD (range): 11.30 (4.72–20.98; ± 0.72) Follow-Up since EOT: mean ± SD: 1.47 ± 0.12	Once during follow-up	3
Bérard, 2020 [28]	Prospective cohort. D, P	Recruited 01/2013–12/2016. N = 241	ALL	4.7 (0.9–18.0) Follow-Up since EOT: 12.9 (3.3–26.1)	Once during follow-up in 5-year survivors	3
Bhatt, 2021 [29]	Prospective cohort (SJLIFE). D, P	Diagnosed 1962–2012 Whole cohort: N = 33 HSCT cohort: N = 66	AML, MDS	Whole cohort: mean ± SD 9.6 ± 6.0; HSCT cohort: mean ± SD: 9.7 ± 5.8 Follow-Up since EOT whole cohort: mean ± SD: 22.4 ± 7.8; HSCT cohort: mean ± SD: 20.6 ± 5.4	Once during follow-up in 10-year survivors	3
Bis, 2022 [30]	Retrospective cohort. D, P	Last follow-up visit 2016–2019. N = 198	ALL, AML, CML, MDS treated with HSCT	Mean ± SD: 9.33 ± 5.29 Follow-Up since HSCT: median ± SD: 3.8 ± 1.8	TP 1: one month before HSCT TP 2: first time of lipid disorder after HSCT TP 3: last follow-up visit	3
Bolier, 2024 [20]	Prospective cross-sectional cohort. D, P	Diagnosed 1963–2002. N = 2338	All types of childhood cancer	0–4 (45.8%), 5–9 (27.8%), 10–14 (20.6%), 15–17 (5.8%) Follow-Up since Dx: 10–20 (20.2%), 20–30 (39.7%), 30–40 (29.6%), 40–50 (9.4%), 50–59 (1.1%)	Once during follow-up	1
Cacciotti, 2021 [31]	Prospective cross-sectional. D, P	Recruited 09/2012–08/2014. N = 12	CNS tumors	Age at evaluation: mean (range): 11 (7–16.7) Follow-Up since HSCT: mean (range) 3.75 (1.5–5.9)	Once during follow-up	2
Cepelova, 2019 [32]	Prospective cross-sectional. D, P	Diagnosed 02/1977–12/2017. Recruited 05/2015–12/2017. N = 80	Hodgkin lymphoma	14.9 (4.5–19.3) Follow-Up (not defined): 19.2 (10–34.6)	Once during follow-up	2
Ciolli, 2024 [33]	Retrospective case report. D, T	2023. N = 1	Lymphoma	25	During treatment	2
Cooksey, 2019 [34]	Prospective cross-sectional. D, P	NR. N = 142	CNS tumors	HPA-irradiated: median (IQR): 7.2 (5.7–9.0); non-HPA-irradiated: median (IQR): 4.9 (3.6–5.6) Follow-Up since EOT in irradiated: median (IQR): 4.5 (3.5–5.5); Follow-Up non-irradiated: median (IQR): 6.3 (4.3–7.4)	Once during follow-up	2
Das, Nutrition and Cancer, 2024 [35]	Prospective cross-sectional. D, P	Recruited 12/2020–12/2021. N = 40	ALL	Median (IQR) 5.9 (3.7–10.6) Follow-Up since Dx: median (IQR): 7.2 (6.1–10.6) Follow-Up since EOT: median (IQR): 5 (3.3–7.5)	Once during follow-up in 2-year survivors	3
Das, Supp Care Cancer, 2024 [36]	Prospective cross-sectional. D, P	Recruited 11/2020–12/2021. N = 65	ALL	Mean ± SD: 12.9 (±3.2) Follow-Up since Dx: median (IQR): 6.5 (5.9–8)	Once during follow-up in 2-year survivors	3
Delorme, 2025 [37]	Not randomized controlled study (VIE study) D, P, T	Diagnosed and recruited 02/2018–12/2019. N = 45	All types of childhood cancer	5.7 (1.3–16.1) Follow-Up since EOT: 1.4 (0.0–3.5)	Once during follow-up	3
England, 2017 [38]	Prospective cohort (PETALE). D, P	Diagnosed 1987–2010. N = 209	ALL	Age <19 years at diagnosis Age at evaluation: 22.4 (8.5–41.0) Follow-Up since Dx: mean ± SD: 15.5 ± 5.2	Once during follow-up in 5-year survivors	2
Faber, 2018 [39]	Prospective cross-sectional (CVSS). P	Recruited 10/2013–02/2016. N = 951	All types of childhood cancer, excl. Hodgkin lymphoma	Mean (range): 6.1 (0–14) Follow-Up since diagnosis: mean (range): 28.4 (23–36)	Once during follow-up in 5-year survivors	2
Fachin, 2023 [40]	Retrospective case report. T	NR. N = 1	ALL	10	Repeatedly during leukemia treatment	1
Felicetti, 2015 [41]	Prospective cohort. D, P, T	1973–2007. N = 330	All types of childhood cancer	Age at Follow-Up: mean ± SD: 24.1 ± 3.2 Follow-Up since EOT: 16.1 (4–33)	At each follow-up visit (cumulative incidence reported)	3
Goldberg, 2024 [22]	Prospective cohort. D, P	Diagnosed 1962–2012. N = 4115	All types of childhood cancer	Mean ± SD: 8.6 ± 5.7 Follow-Up since Dx: mean ± SD: 26.5 ± 10.4	Once during follow-up	2
Heenan, 2021 [42]	Correspondence. D, P, T	NR. N = 4 (N = 2 with lipid measurement)	T-ALL and T-LBL	Both cases 17 years Follow-Up: NR	During treatment	3
Hwang, 2024 [43]	Retrospective cohort. D, P	Diagnosed 01/2000–12/2022. N = 200	Hematologic and solid tumors treated with HSCT	9.9 (0.1–18.0) Follow-Up since HSCT: 14.0 (2.4–24.6)	Once during follow-up	2
Javalkar, 2022 [44]	Retrospective cohort. D, P, T	Recruited 2010–2019. N = 52	All types of childhood cancer	Age at study: median (IQR): 16.1 (13.0–18.7) Follow-Up: NR	At least twice during follow-up	3
Jin, 2023 [45]	Retrospective cross-sectional. D, P	Recruited 01/2013–08/2021. N = 310	All types of childhood cancer	Mean ± SD (range): 11.8 ± 4.95 (0.4–19.9) Follow-Up (not defined): Mean ± SD: 9.2 ± 4.16 (1.0–20.3)	Once during follow-up in 1-year survivors	2
Kaplan, 2024/2025 [46]	Retrospective case report. D, T	NR. N = 1	AML	4	During treatment: before and after fenofibrate	3
Khera, 2022 [47]	Retrospective case report. T	NR. N = 1	ALL	Toddler	Repeatedly during treatment	2
Lau, 2021 [48]	Retrospective case series. T	NR. N = 2	ALL	12 and 13 years	Repeatedly during treatment	2
Laumann, 2021 [49]	Prospective pilot RCT with historical control. T	Recruited 12/2017–06/2018. N = 7	ALL	Median (IQR): 7 (2–10) Follow-Up: day 113 of intervention	Repeatedly during treatment: baseline (day 30); eight times until day 113	3
Lee, 2021 [50]	Retrospective cohort. P	Diagnosed 01/2000–12/2018. N = 253	Non-central nervous system solid tumors	Mean ± SD: 4.9 ± 4.9 Follow-Up (not defined): Mean ± SD: 8.95 ± 4.6	At each annual follow-up visit (cumulative incidence reported)	3
Levy, 2017 [13]	Prospective cohort (PETALE). D, P	Diagnosed 1987–2005. N = 247	ALL	4.7 (0.8–18.0) Follow-Up since Dx: 15.2 (5.4–28.2)	Once during follow-up in 5-year survivors	2
Lubas, 2021 [51]	Prospective cohort (SJLIFE). D, P	NR. N = 3267	All types of childhood cancer	7.7 (0–24.8) Follow-Up: NR	Twice during follow-up in 5-year survivors: baseline and follow-up of median 3.9 years	2
Mayerhofer, 2020 [52]	Case Report. D, T	2020. N = 1	ALL	14	Repeatedly during treatment	2
Mogensen, 2020 [11]	Retrospective cohort. D, P	Diagnosed 07/2008–12/2016. N = 127	ALL	Median (IQR): 4.7 (2.09–10.4) Follow-Up: NR	Repeatedly during treatment: all at diagnosis; not systematically thereafter	1
Mohapatra, 2016 [53]	Prospective observational and cross-sectional. D, P	Recruited 01/2023–12/2013. N = 76	ALL	Median (IQR): 5 (3.3–6.5) Follow-Up since EOT: median (IQR): 3 (2.3–5)	During follow-up in 2-year survivors	3
Morel, 2019 [54]	Prospective cohort (PETALE). D, P	Diagnosed 1987–2005. Recruited 01/2013–12/2016. N = 241	ALL	4.7 (0.9–18.0) Follow-Up since EOT: 12.9 (3.3–26.1)	Once during follow-up in 5-year survivors	1
Nagayama, 2019 [55]	Prospective case report. D, T	NR. N = 1	ALL	3	Before, 1 month and 6 months after Metreleptin	3
Napartuk, 2023 [56]	Not randomized controlled study (VIE study). T	Diagnosed and recruited 02/2018–12/2019. N = 36	All types of childhood cancer	Mean ± SD: 7.88 ± 5.0 Follow-Up since Dx: 1 year	Twice during treatment: at start of intervention and 1 year	3
Nirmal, 2021 [57]	Prospective observational. D, P	Recruited 04/2018–03/2019. N = 277	ALL	Mean ± SD: 5.2 ± 3.2 Follow-Up since EOT: mean (range): 5.4 (2.1–18.5)	Once during follow-up in 2-year survivors	1
Oudin, 2015 [58]	Prospective cohort (LEA study). D, P	Diagnosed since 1980. Recruited 2007–2012. N = 170	ALL, AML	Mean ± SD: 8.6 ± 4.9 Follow-Up since HSCT: mean ±SD: 14.5 ±6.1	Repeatedly during follow-up (cumulative incidence reported)	2
Özdemir, 2018 [59]	Prospective case control study. D, P	Diagnosed 2003–2013. N = 50	ALL	5 (3–11) Follow-Up since EOT: mean ± SD: 4.14 ± 2.48	Once during follow-up	3
Persson, 2017 [60]	Retrospective cohort. D, P	Diagnosed 07/2008–12/2014. N = 92	ALL	4.8 (1.1–17.8) Follow-Up: NR	During treatment, before each asparaginase	2
Pluimakers, 2020 [61]	Prospective cross-sectional. D, P	Diagnosed 1961–2004. N = 103	Nephroblastoma, neuroblastoma	Median (IQR): 2.3 (0.8–5.0) Follow-Up (not defined): median (IQR): 27.5 (20.1–31.6)	Once during follow-up in 5-year survivors	1
Pranjić, 2025 [62]	Retrospective cross-sectional. D, P	Diagnosed 2016–2023. N = 16	Hodgkin lymphoma	Median (IQR): 15.0 (3.0) Follow-Up since Dx: median ± SD: 4.3 ± 2.4	Once during follow-up	2
Salvador, 2018 [63]	Retrospective cohort T	Diagnosed 2000–2009. N = 119	ALL	Mean ± SD (range): 7.48 ± 4.65 (1.7–17.34) Follow-Up: NR	During treatment: weekly after start of leukemia treatment	2
Saultier, 2016 [64]	Prospective cohort (LEA study). D, P	Diagnosed since 1980 Recruited 2007–2013. N = 650	Leukemia	Mean ± SD: 8.22 ± 4.80 Follow-Up since Dx: mean ± SD: 16.00 ± 6.79	Repeatedly during follow-up (cumulative incidence reported for lipids)	1
Schindera, 2021 [65]	Prospective RCT. D, P	Recruited 2015–2019. N = 163	All types of childhood cancer	6.7 (3.1–11.8) Follow-Up since Dx: 22.3 (16.0–29.1)	Once during follow-up (cross-sectional analysis in this publication)	2
Schmidt, 2021 [66]	Retrospective cohort. D, P	Diagnosed 01/2010–12/2019. N = 165	ALL	1–18 years Follow-Up (not defined): 5 (0.1–11.5)	During treatment, not clearly specified	3
Sonowal, 2019 [67]	Retrospective case report. D	NR. N = 1	ALL	1.5	Repeatedly during treatment: baseline at diagnosis, day 3, end of 1st week	2
Warris, 2016 [68]	Prospective RCT. P, T	NR. N = 50	ALL	Median (IQR): 6.0 (4.0–10.3) Follow-Up: 5th day of treatment	Twice during treatment: before dexamethasone (T1), day 5 of treatment (T2)	2
Wei, 2017 [69]	Retrospective cohort. D, P	Recruited 2011–2012. N = 52	ALL	HSCT/TBI group: 5.6 (1.0–10.8) Chemo only group: 7.0 (1.6–18.0) Follow-Up: NR	Once during follow-up in 3-year survivors	2
Zareifar, 2017 [70]	Retrospective cohort. D, P	Diagnosed 07/2012–07/2013. N = 53	ALL	Mean ± SD: 5.9 ± 3.8 Follow-Up (not defined): 3.5 ± 1.5	During treatment and follow-up, TP unclear	3
Zawitowska, 2019 [71]	Retrospective case report. T	NR. N = 2	ALL	5 and 10	Repeatedly during treatment: during treatment, before and after start of antihypertriglyceridemic treatment	2

Abbreviations: ALL, acute lymphoblastic leukemia; AML, acute myeloid leukemia; CML, chronic myeloid leukemia; CVSS study, cardiac and vascular late sequelae in long-term survivors of childhood cancer; EOT, end of treatment; HPA, hypopituitary axis; LEA cohort, Leucémie de l’Enfant et de l’Adolescent; MDS, myelodysplastic syndrome; NR, not reported; RCT, randomized controlled trial; SJLIFE, St. Jude Lifetime Cohort Study; TP, time point; Dx, diagnosis; VIE study, Valorization, Implication, Education. * Study outcome types: D, definition of dyslipidemia; P, prevalence of dyslipidemia; T, treatment. ° Age and follow-up time expressed as a median (range) if not specified.

**Table 2:** Definition of abnormal lipid parameters, comparing the definition used in adults and children.

**Triglycerides**	
**Adults**	**Children**
>1.56 mmol/L (139 mg/dL) [55]	NA
≥1.7 mmol/L (≥150 mg/dL) [13]	≥1.47 mmol/L [13]
≥1.7 mmol/L (≥150 mg/dL) [20,22,23,25,26,30,31,32,33,34,35,36,38,42,45,46,55,57,58,59,61,62,65,72]	Same cutoff as for adults or if the cohort included adult survivors only
>1.7 mmol/L (>150 mg/dL) [27]	>1.12 mmol/L (>99 mg/dL) (0–9 years) >1.46 mmol/L (>129 mg/dL) (≥10 years) [27]
>1.7 mmol/L (>2.26 mmol/L (>200 mg/dL); >4.40 mmol/L (390 mg/dL)) [37,41,43,67]	Same cutoff as for adults or if the cohort included adult survivors only
	>2.69 mmol/L (>236 mg/dL) [11]
≥2.25 mmol/L (199 mg/dL) [43]	≥1.47 mmol/L (≥ 130 mg/dL) [43]
NA	≥1.13 mmol/L (≥100 mg/dL) (<9 years) [44] ≥ 1.47 mmol/L (≥ 130 mg/dL) (≥9 years) [44]
≥1.7 mmol/L (≥150 mg/dL) [25,28,54]	≥1.13 mmol/L (≥100 mg/dL) (<10 years) [25,28,54] ≥1.46 mmol/L (>129 mg/dL) (≥10 years) [25,28,54]
NA	≥5× age-dependent upper normal limit (UNL): 1–5 years: >5.0 mmol/L (443 mg/dL); 6–11 years: >6 mmol/L (531 mg/dL); 12–15 years: >8 mmol/L (709 mg/dL); >16 years: >9 mmol/L (797 mg/dL) [60]
Modified CTCAE Grade 1–4 * [29,51]	NA
CTCAE version 5.0, grade ≥3 (>500 mg/dL or > 5.7 mmol/L) [66]	NA
**LDL-C**	
**Adults**	**Children**
≥3.36/3.4 mmol/L (130 mg/dL/132 mg/dL) [55,62]	NA
≥3.36/3.4 mmol/L (≥130 mg/dL/132 mg/dL)) [13,26,28,37,38,44,45,54,59]	Same cutoff as for adults or if the cohort included adult survivors only [13,26,28,37,38,44,45,54,59]
≥4.13 mmol/L (160 mg/dL) [22,41,67]	NA
≥4.13 mmol/L (160 mg/dL) [27]	≥3.36 mmol/L (≥130 mg/dL) [27]
NA	>2.55 mmol/L (99 mg/dL) (Girls 1–6 years) >2.81 mmol/L (109 mg/dL) (Boys 1–6 years) >3.4 mmol/L (132 mg/dL) (Children >6–19 years) [11]
≥3.4 mmol/L (≥132 mg/dL) [37]	>2.85 mmol/L (110 mg/dL) [37]
**HDL-C**	
**Adults**	**Children**
<1.03 mmol/L [24,25,26,27,30,35,36,37,43,44,54,55,59,67]	Same cutoff as for adults or if the cohort included adult survivors only [24,25,26,27,30,35,36,37,43,44,54,55,59,67]
<1.03 mmol/L (<40 mg/dL) (men) <1.3 mmol/L (<50 mg/dL) (women) [20,22,23,32,38,45,51,57,58,61,62,65,69,72]	Same cutoff as for adults or if the cohort included adult survivors only [20,22,23,32,38,45,51,57,58,61,62,65,69,72]
<1.03 mmol/L (<40 mg/dL) (men) <1.3 mmol/L (<50 mg/dL) (women) [13,28,34,54,57]	<1.03 mmol/L (<40 mg/dL) [13,28,34,54,57]
<1.0 mmol/L (39 mg/dL) (Men) <1.3 mmol/L (50 mg/dL) (Women) [31]	<0.91 mmol/L (35 mg/dL) (2–10 years) <1.0 mmol/L (39 mg/dL) (10–16 years) [31]
NA	<0.8 mmol/L (31 mg/dL) (1–4 years) <0.9 mmol/L (35 mg/dL) (4–6 years) <1.0 mmol/L (39 mg/dL) (Boys 6–14 years) <0.8 mmol/L (31 mg/dL) (Boys 14–19 years) <1.0 mmol/L (39 mg/dL) (Girls 6–19 years) [11]
NA	≤5th percentile [70]
**Total Cholesterol**	
**Adults**	**Children**
Lower values than ≥5.17 mmol/L (200 mg/dL) ((>4.4 mmol/L (170 mg/dL); >5.0 mmol/L (193 mg/dL)) [62,67]	NA
≥5.17 mmol/L (≥200 mg/dL) [24,26,33,37,41,44,45,54,59]	Same cutoff as for adults or if the cohort included adult survivors only [24,26,33,37,41,44,45,54,59]
≥5.79 mmol/L (≥224 mg/dL) [27]	≥5.79 mmol/L (≥224 mg/dL) [27]
≥6.2 mmol/L (≥240 mg/dL) [43]	≥6.2 mmol/L (≥240 mg/dL) [43]
Higher values than ≥5.17 mmol/L (≥200 mg/dL) (>5.5 mmol/L (213 mg/dL); >5.68 mmol/L (220 mg/dL) [42,46,55]	NA
	>5.1 mmol/L (Girls <3 years) >5.7 mmol/L (Boys < 3 years) >6.1 mmol/L (Girls 3–6 years) >6.0 mmol/L (Boys 3–6 years) >5.5 mmol/L (Children 6–19 years) [11]
Modified CTCAE Grade 1–4 * [29,51]	NA

Abbreviations: NA, not included/reported in the cohort. * High total cholesterol acc. to modified CTCAE criteria [73]: Grade 1: >200– 300 mg/dL; Grade 2: >300–400 mg/dL or treatment with one lipid-lowering agent; Grade 3: >400–500 mg/dL or treatment with ≥2 lipid-lowering agents; Grade 4: >500 mg/dL.

**Table 3:** Prevalence of abnormal lipid values and dyslipidemia.

Range	Values	Mean Value
**High triglycerides**		
<20%	2.4% [50]; 4% [59]; 6% [41]; 6.7% [66]; 8.6% [34]; 10% [69]; 11.7% [64]; 12% [27]; 12.2% [13,54]; 17% [31], 17.2% [22]; 19% [65]; 19.7% [53]	29%
20% to <40%	20.9% * [29] 23% [61]; 24.9% [57]; 25% [32]; 26% [23,36]; 26.8% [45]; 27.4% [20]; 29.6% [58]; 32% [70]; 34% [63]	
40% to <60%	45.4%° [29]; 48% [69]; 58% [11]	
60% to <80%	65% [60]	
≥80%	85% [24]; 82.9% [30]; 85.3% [44]	
**High LDL-C**		
<20%	1% [11]; 2.1% [50]; 6% [59] 8.7% [22] 10% [27]; 11.7% [37]; 17.4% [13,54]	18%
20% to <40%	37.6% [30]	
40% to <60%	40% [36]; 47.6% [44]	
**Low HDL-C**		
<20%	14% [59]; 14.6% [34]; 16% [27]; 17.5% [32]; 18% [65]; 19.5% [37]	35%
20% to <40%	23.1% [13,54]; 25% [31,34]; 25.4% [45]; 26.8% [64]; 27% [69]; 28.2% [57]; 29% [61]; 36.4% [20,58]; 36.8% [23,53]	
40% to <60%	44.4% [62]; 45.2% [70]; 57% [69]	
60% to <80%	60.5% [22]; 62.5% [44]; 62.9% [30]	
≥80%	99% [11]	
**High Total Cholesterol**		
<20%	0% [24]; 4% [59]; 5% [11]; 10% [27]; 11.7% [37]	31%
20% to <40%	20% [41]	
40% to <60%	46.9% [29]	
60% to <80%	62.8% [44]; 75% [30,31]	
**Dyslipidemia**		
<20%	3.2% [50]; 12.7% [51]	34%
20% to <40%	22% [43]; 23.3% [26]; 23.9% [29]; 25% [31]; 28.3% [39]; 30% [27]; 38% [59]	
40% to <60%	41.1% [28]; 41.3% [13]; 41.4% [54] 41.8% [38]	
60% to <80%	-	
≥80%	99% [11]	
**Prevalence in one study with longitudinal data (intervention group)**		
High TGs	Baseline: 85%; 3 months follow-up: 50% [24]	
High Total Cholesterol	Baseline: 0%; 3 months follow-up: 0% [24]	

Bhatt et al. [29] reports the prevalence in survivors with HSCT * and without HSCT/conventional therapy.

**Table 4:** Overview of studies to promote normal lipid values (prevention and management).

Author, Timepoint	Diagnosis	Age [Years]	Intervention and Its Duration	Results
**Barbosa-Cortés et al.** [24] During treatment	Acute lymphoblastic leukemia	Mean ± SD: 6.7 ± 2.7	Intervention: Omega3-LCPUF capsules; administered as 500 mg soft capsules of natural TG containing 225 mg DHA, 5 mg EPA, and 20 mg of another w3-LCPUFAs at a rate of 0.100 g/kg of body weight/day Duration: first three months of leukemia treatment	High TG intervention vs. placebo at baseline: 85% vs. 80% High TG intervention vs. placebo at 3 months: 50% vs. 85% High total cholesterol intervention vs. placebo at baseline: 0% vs. 0% High total cholesterol intervention vs. placebo at 3 months 0% vs. 0%
**Delorme et al. [37]** During treatment	All types of childhood cancer	5.7 (1.3–16.1)	Intervention 1 (nutrition): Promote healthy eating behaviors Intervention 2 (physical activity): 1) Baseline assessment to evaluate physical capacity; 2) individualized training program; 3) 2–3 physical activity sessions of 45 min per week over 6 weeks; 4) regular reassessment of individualized program Intervention 3 (psychological): Psychological intervention offered to the parents; 6 × 60 min Duration: 1 year with follow-up visits every 2 months from baseline	High cholesterol in intervention vs. control group at 15 months post-treatment: 11.9% vs. 11.7%, *p* = 1.0 High LDL-C control in intervention vs. control group at 15 months post-treatment: 9.8% vs. 11.7%, *p* = 0.773 Low HDL-C control in intervention vs. control group at 15 months post-treatment: 19.5% vs. 19.5%, *p* = 1.0
**Javalkar et al. [44]** During survivorship	All types of childhood cancer	Age at study: median (IQR): 16.1 (13–18.7) Follow-Up: NR	Intervention 1: Guidance on lifestyle modifications: (1) preparation of a clinical management plan based on the 2011 National Heart, Lung, and Blood Institute (NHLBI) Expert Panel on Integrated Guidelines for Cardiovascular Health and Risk Reduction in Children and Adolescents (add reference); (2) Risk stratification; (3) Individualized guidance on lifestyle modifications (e.g., diet, physical activity); (4) Reassessment at follow-up visit Intervention 2: Pharmacotherapy initiated if NHLBI guideline-defined thresholds not met after 6 months of monitoring with lifestyle changes alone (drugs not specified) Duration: Not stated; at least one follow-up visit	Absolute change in total cholesterol in survivors vs. controls: median −16.6 mg/dL (IQR −34.5–2.5) vs. median −13.1 (IQR −28.9–7.7), *p* = 0.667 Absolute change in triglyceride in survivors vs. controls: median −72.5 mg/dL (IQR −119.5–−1.6) vs. median −17 (IQR −98.5–0), *p* = 0.095 Absolute change in HDL-C survivors vs. controls: median 1.9 mg/dL (IQR 0–7) vs. median 1 (IQR −1–3), *p* = 0.304 Absolute change in LDL-C in survivors vs. controls: median -4 mg/dL (IQR −40–4) vs. median −7 (IQR −27–10), *p* = 0.441
**Laumann et al. [49]** During treatment	Acute lymphoblastic leukemia	Median (IQR): 7 (2–10)	Intervention 1: 10- or 20-mL fish oil containing 2.4–4.8 g EPA + DHA daily Duration: Between day 30 and day 113 of leukemia treatment	Median (IQR) TG on day 113 in intervention vs. control: 1.19 mmol/L (0.87–2.57) vs. 3.15 mmol/L (2.13–6.30); *p* = 0.186 Median (IQR) total cholesterol on day 113 in intervention vs. control: 4.5 mmol/L (3.60–8.08) vs. 7 mmol/L (5.00–10.15), *p* = 0.08
**Napartuk et al. [56]** During treatment	All types of childhood cancer	Mean ± SD: 7.88 ± 5.0 follow-up since diagnosis: 1 year	Intervention 1: Individualized nutritional assessment and nutritional counselling: (1) initial assessment; (2) follow-up visits every 2 months; (3) 1-year assessment Duration: 1 year with follow-up visits every 2 months from baseline	Mean ± SD total cholesterol at baseline and one year: 4.08 ± 2.03 and 4.01 ± 1.04; *p* = 0.63 Mean ± SD HDL-C at baseline and one year: 0.94 ± 0.29 and 1.22 ± 0.29, *p* = 0.002 Mean ± SD LDL-C at baseline and one year: 2.62 ± 1.89 and 2.28 ± 1.07, *p* = 0.22

Abbreviations: DHA, Docosahexaenoic acid; EPA, Eicosapentaenoic acid; LCPUF, long-chain polyunsaturated omega-3 fatty acids; IQR, interquartile range; NHLBI, National Heart, Lung, and Blood Institute; SD, standard deviation.

## Data Availability

The original contributions presented in this study are included in the article/Supplementary Material. Further inquiries can be directed to the corresponding author.

## Supplementary Materials

The following supporting information can be downloaded at: https://www.mdpi.com/article/10.3390/cancers18050837/s1, Supplementary Table S1: Search strategy; Supplementary Table S2: Definitions of dyslipidemia sorted by first authors; Supplementary Table S3: Other definitions of dyslipidemia by publication; Supplementary Table S4: Outcomes assessed and prevalence of abnormal values, reported by first authors; Supplementary Table S5: Results from case reports and small case series in interventions, all performed during treatment and at diagnosis of dyslipidemia; Supplementary Table S6: Critical appraisal of studies included in the final systematic review, according to JBI.

## Abbreviations

The following abbreviations are used in this manuscript:

ALL	Acute Lymphoblastic Leukemia
AML	Acute Myeloid Leukemia
BMI	Body Mass Index
CAYA	Childhood, Adolescent and Young Adult
CI	Confidence Interval
CVD	Cardiovascular Diseases
HDL-C	High-Density Lipoprotein Cholesterol
JBI	Joanne Briggs Institute
IDF	International Diabetes Federation
IQR	Interquartile Range
LDL-C	Low-Density Lipoprotein Cholesterol
MetS	Metabolic Syndrome
NR	Not Reported
OR	Odds Ratio
RCT	Randomized Controlled Trial
RT	Radiotherapy
TC	Total Cholesterol
TG	Triglyceride
VLDL	Very-Low Density Lipoprotein
