# Supplementary material for: Definition, Prevalence and Management of Dyslipidemia in Patients and Survivors of Childhood and Adolescent Cancer—A Systematic Review
Source: Cancers (Basel). 2026 Mar 4;18(5):837. doi: 10.3390/cancers18050837 (PMC12984902; doi:10.3390/cancers18050837)
Supplement: Supplementary file 1 [file cancers-18-00837-s001.zip › cancers-4121397-supplementary.pdf]

cancers

Definition, Prevalence and Management of Dyslipidemia in Patients and Survivors of Childhood and Adolescent Cancer—A Systematic Review

Fiona L Wagenseil [1], Luca Bühlmann [1], Stephanie B Dixon [2,3], Matthew J Ehrhardt [2,3], Sarah P Schladerer [1], Cornelia Vetter [4], Maria Otth [4,5,*,†] and Katrin Scheinemann [1,4,†]

[1] Faculty of Health Sciences and Medicine, University of Lucerne, 6002 Lucerne, Switzerland
[2] Department of Epidemiology and Cancer Control, St. Jude Children's Research Hospital, Memphis, TN 38105-3678, USA
[3] Department of Oncology, St. Jude Children's Research Hospital, Memphis, TN 38105-3678, USA
[4] Division of Oncology-Hematology, Children's Hospital of Eastern Switzerland, 9006 St. Gallen, Switzerland
[5] Department of Oncology, University Children's Hospital Zurich-Eleonore Foundation, 8008 Zurich, Switzerland
* Correspondence: maria.otth@kispisg.ch
† These authors contributed equally to this work.

Supplementary Material

Supplementary Table S1: Search strategy

**P**	1. Cancer diagnoses in CAYA cancer patients	Leukemia[TiAb] OR leukemi*[TiAb] OR leukaemia[TiAb] OR leukaemi*[TiAb] OR lymphoma[TiAb] OR lymphom*[TiAb] OR hodgkin[TiAb] OR hodgkin*[TiAb] OR non-hodgkin[TiAb] OR non-hodgkin*[TiAb] OR sarcoma[TiAb] OR sarcom*[TiAb] OR sarcoma, Ewing's[TiAb] OR Ewing*[TiAb] OR osteosarcoma[TiAb] OR osteosarcom*[TiAb] OR wilms tumor[TiAb] OR wilms*[TiAb] OR nephroblastom*[TiAb] OR neuroblastoma[TiAb] OR neuroblastom*[TiAb] OR rhabdomyosarcoma[TiAb] OR rhabdomyosarcom*[TiAb] OR teratoma[TiAb] OR teratom*[TiAb] OR hepatoma[TiAb] OR hepatom*[TiAb] OR hepatoblastoma[TiAb] OR hepatoblastom*[TiAb] OR PNET[TiAb] OR PNET*[TiAb] OR medulloblastoma[TiAb] OR medulloblastom*[TiAb] OR neuroectodermal tumors, primitive[TiAb] OR retinoblastoma[TiAb] OR retinoblastom*[TiAb] OR meningioma[TiAb] OR meningiom*[TiAb] OR glioma[TiAb] OR gliom*[TiAb] OR "pediatric oncology"[TiAb] OR "paediatric oncology"[TiAb] OR childhood cancer[TiAb] OR childhood tumor[TiAb] OR childhood tumors[TiAb] OR "brain tumor*"[TiAb] OR "brain tumour*"[TiAb] OR brain neoplasms[TiAb] OR ("central nervous system"[TiAb] AND neoplasm[TiAb]) OR ("central nervous system"[TiAb] AND neoplasms[TiAb]) OR ("central nervous system"[TiAb] AND tumor*[TiAb]) OR ("central nervous system"[TiAb] AND tumour*[TiAb]) OR "brain cancer*"[TiAb] OR "brain neoplasm*"[TiAb] OR "intracranial neoplasm*"[TiAb]
**P**	2. Different age categories (infants, children, adolescents, young adults)	Infan*[TiAb] OR toddler*[TiAb] OR minors[TiAb] OR minors*[TiAb] OR boy[TiAb] OR boys[TiAb] OR boyfriend[TiAb] OR boyhood[TiAb] OR girl*[TiAb] OR kid[TiAb] OR kids[TiAb] OR child[TiAb] OR child*[TiAb] OR children*[TiAb] OR schoolchild*[TiAb] OR schoolchild[TiAb] OR school child[TiAb] OR school child*[TiAb] OR adolescen*[TiAb] OR juvenil*[TiAb] OR youth*[TiAb] OR teen*[TiAb] OR under*age*[TiAb] OR pubescen*[TiAb] OR pediatrics[mh] OR pediatric*[TiAb] OR paediatric*[TiAb] OR peadiatric*[TiAb] OR school[TiAb] OR school*[TiAb] OR young adult[mh] OR "young adult*"[TiAb]
	3. Combine	#1 AND #2
**I**	4. Dyslipidemia	dyslipidaemia OR dyslipidemia OR ("low* cholesterol") OR ("high* cholesterol") OR ("elevated cholesterol") OR ("high* triglycerid*") OR ("elevated triglycerid*") OR ("low* HDL") OR ("high* LDL") OR ("elevated LDL") OR hyperlipidemia OR hyperlipidaemia OR hyperlipidemias[MeSH] OR hyperlipidemia* OR hypercholesterolemia OR hypercholesterolaemia OR hypertriglyceridemia OR hypertriglyceridaemia OR "metabolic syndrom*" OR metabolic syndrome[MeSH] OR "insulin-resistance syndrome" OR "syndrom* X" OR cardiometabolic
**I**	5. Treatment	treatment OR intervention OR medication OR drug OR counseling OR ("lifestyle modification") OR ("cholesterol lower*") OR (lipid-lowering agent*) OR antihyperlipidemic OR fibric acids[MeSH] OR fibrate* OR fibric acid* OR fenofibric acid* OR gemfibrozil OR gemfibrocil OR bezafibrate OR clofibrate OR fenofibrate OR statin* OR fluvastatin OR simvastatin OR pravastatin OR lovastatin OR meglutol OR cerivastatin OR atorvastatin OR cholestyramine* OR "vitamin B3" OR niacin* OR ezetimibe OR 2-Azetidione

		OR “bile acid sequester*” OR cholestyramine OR colesevelam OR colestipol OR “Adenosine triphosphate-citrate lyase” OR “ANGPTL3 inhibit*” OR evinacumab OR “PCSK9 inhibit*” OR alirocumab OR evolocumab OR "MTP inhibit*" OR lomitapide OR “omega-3”
	6. Combine	#4 AND #5
	7. Combine	#3 AND #6
C	*Comparators*	None
O	*Outcome*	None
	8. Humans only	animals[mh] NOT humans[mh]
	9. Combine	#7 NOT #8
	10. Date restriction	"2015/01/01"[Date - Publication]: "2025/02/01"[Date - Publication]
	12. Combine	#9 AND #10

Supplementary Table S2: Definitions of dyslipidemia sorted by first authors

First Author	Definition of dyslipidemia
Armstrong [1]	TG ≥150 mg/dL or treatment for elevated TG<br>HDL-C <40 mg/dL (men) / <50 mg/dL (women)
Barbosa-Cortes [2]	TG<br>- acceptable <75 mg/dL, borderline 75-99 mg/dL, high ≥100mg/dL (children <10 years)<br>- acceptable <90 mg/dL, borderline 90-129 mg/dL, high ≥130 mg/dL<br>HDL-C <40 mg/dL<br>Total Cholesterol acceptable <170 mg/dL / borderline 170-199 mg/dL / high ≥200 mg/dL
Barbosa-Cortes [3]	TG ≥150 mg/dL<br>HDL-C <40 mg/dL
Bayram [4]	Total Cholesterol >200 mg/dL<br>TG >150 mg/dL<br>LDL-C >130 mg/dL<br>HDL-C <40 mg/dL<br>Dyslipidemia if any of the above-mentioned parameters being abnormal
Bélanger [5]	TG >1.12 mmol/L (0-9 years), >1.46 mmol/L (>10 years), >1.69 mmol/L (20-24 years)<br>Total Cholesterol (TC) >5.17 mmol/L (0-19 years) or 5.79 mmol/L (20-24 years)<br>Free Cholesterol, esterified Cholesterol (EC) [calculated (TC-FC) x1.6]<br>HDL-C <1.03 mmol/L (independent of age)<br>LDL-C ≥3.36 mmol/L (0-19 years) and ≥4.14 mmol/L (> 19 years)<br>Dyslipidemia if high LDL-C, high TG, and/or low HDL-C
Bérard [6]	TG ≥1.7 mmol/L (150.6 mg/dL) in adults, ≥1.47 mmol/L (130.2 mg/dL) in children<br>LDL-C ≥3.4 mmol/L (131.5 mg/dL) in adults, ≥3.36 mmol/L (129.3 mg/dL) in children<br>HDL-C <1.03 mmol/L (39.8 mg/dL) (men), <1.3 mmol/L (50.3 mg/dL) (women), <1.03 mmol/L (39.8 mg/dL) (children)
Bhatt [7]	Hypertriglyceridemia, hypercholesterolemia acc. to CTCAE Grade 1-4
Bis [8]	TG >150 mg/dL<br>HDL-C <40 mg/dL
Bolier [9]	TG >1.7 mmol/L<br>HDL-C <1.0 mmol/L (men)/ <1.3 mmol/L (women)
Cacciotti [10]	TG ≥1.71 mmol/L<br>HDL-C<br>- <0.91 mmol/L (children 2-10 years)

	- <1.0 mmol/L (children 10-16 years)<br>- <1.0 mmol/L (boys) or < 1.3 mmol/L (girls) (children > 16 years)<br>Dyslipidemia if high TG or low HDL-C or lipid lowering agent
Cepelova [11]	LDL-C >3.0 mmol/L<br>HDL-C <1.0 mmol/L<br>TG >1.7mmol/L<br>Dyslipidemia if ≥1 of LDL-C, HDL-C, or TG abnormal
Ciolli [12]	TG >150 mg/dL<br>Total Cholesterol >200 mg/dL
Cooksey [13]	TG >150 mg/dL<br>HDL-C <40 mg/dL for men and women <15 years / men >16 years, < 50mg/dL for women >16 years
Das [14]	TG >150 mg/dL<br>HDL <40 mg/dL
Das [15]	TG >150 mg/dL<br>HDL <40 mg/dL
Delorme [16]	Total Cholesterol: acceptable <4.40 mmol/L, borderline 4.40-5.17 mmol/L, high ≥5.18 mmol/L<br>LDL-C: acceptable <2.85 mmol/L, borderline 2.85-3.35 mmol/L, high ≥3.36 mmol/L<br>HDL-C: low <1.03 mmol/L, acceptable >1.16 mmol/L, borderline 1.03-1.16
England [17]	LDL-C ≥3.4 mmol/L in adults<br>High TG ≥1.7 mmol/L in adults<br>HDL-C <1.03mmol/L (men) / <1.3 mmol/L (women)<br>Dyslipidemia if high LDL-C, high TG and/or low HDL-C
Faber [18]	No definition
Fachin [19]	No definition
Felicetti [20]	Total Cholesterol >200 mg/dL<br>TG >200 mg/dL<br>LDL-C >160 mg/dL
Goldberg [21]	LDL-C >160 mg/dL<br>Non-HDL >190 mg/dL<br>HDL-C < 40mg/dL (men) / <50mg/dL (women)<br>TG >150mg/dL
Heenan [22]	Total Cholesterol >5.5 mmol/L<br>TG >1.8 mmol/L
Hwang [23]	Children<br>- Total Cholesterol ≥200 mg/dL

	- LDL-C ≥130 mg/dL<br>- TG ≥130 mg/dL<br>- HDL-C <40 mg/dL<br>Adults<br>- Total Cholesterol ≥240 mg/dL<br>- LDL-C ≥160mg/dL<br>- TG ≥200mg/dL<br>- HDL-C <40 mg/dL<br>Dyslipidemia if at least one of the above-mentioned values is abnormal
Javalkar [24]	LDL-C ≥130 mg/dL<br>TG ≥100 mg/dL (children <9 years), >130 mg/dL (children ≥9 years)<br>HDL-C ≤40 mg/dL<br>Total Cholesterol ≥200 mg/dL
Jin [25]	Total Cholesterol ≥200mg/dL<br>TG ≥150mg/dL<br>HDL-C <40mg/dL (men), <50mg/dL (women)<br>LDL-C ≥130 mg/dL<br>Dyslipidemia if any of the above-mentioned parameters abnormal
Kaplan [26]	Total Cholesterol >220 mg/dL<br>Triglyceride level ≥150mg/dL
Khera [27]	No definition
Lau [28]	No definition
Laumann [29]	No definition
Lee [30]	No definition
Levy [31]	Adults<br>- LDL-C borderline ≥2.6 mmol/L and <3.4 mmol/L, high ≥3.4 mmol/L<br>- TG borderline ≥1.3 mmol/L and < 1.7 mmol/L, high ≥1.7 mmol/L<br>- HDL-C low < 1.03 mmol/L (men), <1.3 mmol/L (women)<br>Children<br>- LDL-C borderline ≥ 2.85 mmol/L and < 3.36 mmol/L, high ≥ 3.36 mmol/L<br>- TG borderline ≥ 1.00 mmol/L and < 1.47 mmol/L, high ≥ 1.47 mmol/L<br>- HDL-C <1.03 mmol/L<br>Dyslipidemia if high LDL-C, high TG, low HDL-C and/or on drug treatment
Lubas [32]	Definition CTCAE v4.03, grades 2–4<br>- Total Cholesterol grade 2: >300 - 400 mg/dL; or treatment with one lipid lowering agent

	- Total Cholesterol grade 3: >400 - 500 mg/dL; or treatment with ≥2 lipid lowering agent<br>- Total Cholesterol grade 4: >500 mg/dL<br>- TG grade 2: >300 mg/dL - 500 mg/dL; or treatment with one lipid lowering agent<br>- TG grade 3: >500 mg/dL - 1000 mg/dL; or treatment with ≥2 lipid lowering agents<br>- TG grade 4: >1000 mg/dL; life-threatening consequences<br>Definition NCEP-ATP III<br>- HDL-C <40 mg/dL (men), <50 mg/dL (women) or on current medication<br>- TG ≥150 mg/dL or on current medication
Mayerhofer [33]	Severe hypertriglyceridemia > 5’000 mg/dL
Mogensen [34]	Dyslipidemia: Levels above the ULN or below the LLN in one or more of the lipid parameters<br>Cutoff values for mild, moderate, severe elevation in TG and total Cholesterol<br>- mild: 1 - 10x UNL, moderate: 10 - 20x UNL, severe: >20x UNL<br>TG based on Danish Society of Clinical Biochemistry (DSCB, normal range)<br>- 0.48 – 2.69 mmol/L (children 1 < 19 years)<br>Total Cholesterol based on DSCB (normal range)<br>- 1.9 - 5.1 mmol/L for girls; 2.8 - 5.7 mmol/L for boys (children 1 < 3 years)<br>- 3.2 - 6.1 mmol/L for girls; 2.9 - 6.0 mmol/L for boys (children 3 < 6 years)<br>- 2.7 - 5.5 mmol/L both (children 6 < 19 years)<br>LDL-C based on DSCB (normal range)<br>- 0.69 - 2.55 mmol/L for girls; 0.83 – 2.1 mmol/L for boys (children 1 < 6 years)<br>o - 3.4 mmol/L (children 6 < 19 years)<br>HDL-C based on DSCB (normal range)<br>- - 1.6 mmol/L (children 1 < 4 years)<br>- 0.9 - 1.9 mmol/L (children 4 < 6 years)<br>- - 2.3 mmol/L (boys 6 < 14 years)<br>- 0.8 - 2.0 mmol/L (Boys 14 < 19 years)<br>- Girls 6 < 19 years 1.0 - 2.3 mmol/L
Mohapatra [35]	No definition
Morel [36]	HDL-C in adults<br>- <1.03 mmol/L (men), <1.3 mmol/L (women)<br>Children: *Expert Panel on Integrated Guidelines for Cardiovascular Health and Risk Reduction in Children and Adolescents; National Heart; Lung; Blood Institute. Expert panel on integrated guidelines for cardiovascular health and risk reduction in children and adolescents: Summary report. Pediatrics 2011*<br>- HDL-C<br>- Low <40 mg/dL, acceptable <45 mg/dL, borderline-high 40-45 mg/dL

	- TG<br>- Acceptable: < 75 mg/dL (children 0-9 years), < 90 mg/dL (children 10-19 years)<br>- Borderline: 75-99 mg/dL (children 0-9 years), 90 - 129 mg/dL (children 10-9 years)<br>- High: ≥100 mg/dL (children 0-9 years), ≥130 mg/dL (children 10-19 years)<br>- LDL-C<br>- Acceptable: <110 mg/dL, borderline-high: 110-129 mg/dL, high ≥130 mg/dL<br>- Total Cholesterol<br>- Acceptable: <170 mg/dL, borderline-high: 170-199 mg/dL, high ≥200 mg/dL<br>Dyslipidemia: at least one abnormal lipid value
Nagayama [37]	Total Cholesterol ≥140 mg/dL<br>LDL-C >104 mg/dL<br>HDL-C >7.5 mg/dL<br>TG ≥150 mg/dL<br>Apolipoprotein subgroups (normal ranges): A-I: 24.6 -33.3 mg/dL, A-II: 69-105 mg/dL, B: 1.5-3.8 mg/dL, C-II: 5.4-9.0 mg/dL, C-III: 164-284 mg/dL, E: >36 mg/dL
Napartuk [38]	No definition
Nirmal [39]	HDL-C <40 mg/dL (men), <50 mg/dL (women), ≤40 mg/dL (children)<br>TG ≥150 mg/dL (adults), ≥110 mg/dL (children)
Oudin [40]	HDL-C <40 mg/dL (men), <50 mg/dL (women)<br>TG ≥150 mg/dL or on drug treatment for elevated TG
Özdemir [41]	HDL-C ≤40 mg/dL<br>TG ≥150 mg/dL<br>LDL-C ≥130 mg/dL<br>Total Cholesterol ≥200 mg/dL
Persson [42]	Hypertriglyceridemia - defined as triglycerides ≥5× age-dependent upper normal limit (UNL). Additional analysis at ≥10× UNL.<br>Reference <1.0 mmol/L, hypertriglyceridemia if >5.0 mmol/L (children 1-5 years)<br>Reference <1.2 mmol/L, hypertriglyceridemia if >6 mmol/L (children 6-11 years)<br>Reference <1.6 mmol/L, hypertriglyceridemia if >8 mmol/L (children 12-15 years)<br>Reference <1.8 mmol/L, hypertriglyceridemia if >9 mmol/L (children 16+ years)
Pluimakers [43]	TG ≥1.7 mmol/L or use of statins<br>HDL-C ≤1.03 mmol/L (men), 1.29 mmol/L (women) or use of statins<br>LDL-C, apolipoprotein(apo)-A1, apoB, leptin, adiponectin, lipoprotein(a) (Lp(a)) (no cutoff)<br>(NCEP-ATPIII classification)
Pranjić [44]	TG >1.7 mmol/L<br>HDL-C <1.03 mmol/L (men), <1.29 mmol/L (women)

	LDL-C >3.0 mmol/L<br>Total Cholesterol >5.0mmol/L
Salvador [45]	No definition
Saultier [46]	HDL-C <1.03 mmol/L (men), <1.3 mmol/L (women)<br>TG ≥1.7 mmol/L or treatment for hypertriglyceridemia<br>Combination of low HDL-C and elevated TG
Schindera [47]	HDL-C <1.03 mmol/L (men) / <1.30 mmol/L (women) and/or treatment<br>TG ≥1.7 mmol/L and/or treatment
Schmidt [48]	According to CTCAE version 5.0: Hypertriglyceridemia grade ≥ 3<br>- TG grade 3: >500 mg/dL - 1000 mg/dL; or treatment with ≥2 lipid lowering agents<br>- TG grade 4: >1000 mg/dL; life-threatening consequences
Sonowal [49]	Total Cholesterol ≥170 mg/dL<br>TG >200 mg/dL<br>HDL-C <35 mg/dL<br>LDL-C >160 mg/dL<br>VLDL-C >45 mg/dL
Warris [50]	No definition
Wei [51]	TG ≥1.7 mmol/L or lipid-lowering therapy<br>HDL-C <1.03 mmol/L (men), <1.29 mmol/L (women) or lipid-lowering therapy
Zareifar [52]	HDL-C ≤5th percentile (assessed as a component of MS)<br>Triglyceride (TG) ≥95th percentile (assessed as a component of MS)
Zawitowska [53]	No definition

Abbreviations: DSCB, Danish Society of Clinical Biochemistry; HDL-C, high-density lipoprotein; LDL-C, low-density lipoprotein; TG, triglyceride

Supplementary Table S3: Other definitions of dyslipidemia by publication

	**Definition**
Barbosa-Cortés [2]	AIP (atherogenic index in plasma - logarithmic transformation of the TGs/HDL-C ratio)<br>low: < 0.1, borderline: 0.1-0.24, high > 0.24
Bayram, Bélanger, Cacciotti, Cepelova, England, Hwang, Jin, Levy, Mogensen, Morel, Saultier [4,5,10,11,17,23,25,31,34,36,46]	Dyslipidemia defined as at least one abnormal lipid value (TG, HDL-C, LDL-C, total Cholesterol)
Cacciotti [10], Levy [31]	Use of lipid lowering agents
Nagayama [37]	Apolipoprotein subgroups (A-I, A-II, B, C-II, C-III, E)
Sonowal [49]	VLDL-C

Abbreviations: HDL-C, high-density lipoprotein; LDL-C, low-density lipoprotein; TG, triglyceride, VLDL-C, very low density lipoprotein

Supplementary Table S4: Outcomes assessed and prevalence of abnormal values, reported by first authors

First Author	Outcomes assessed	Prevalence of abnormal values
Armstrong [1]	HDL-C, TG	High HDL-C: 36.8%<br>High TG: 26%
Barbosa-Cortes [2]	Total Cholesterol, LDL-C, HDL-C, TG	High TG in intervention vs. placebo at baseline: 85 % vs. 80 %<br>High TG in intervention vs. placebo at 3 months: 50 % vs. 85 %<br>High total Cholesterol in intervention vs. placebo at baseline and at 3 months: 0 % vs. 0 %<br>HDL-C, LDL-C, VLDL-C, and AIP: NR
Bayram [4]	Total Cholesterol, LDL-C, HDL-C, TG, dyslipidemia	Abnormal Total Cholesterol, TG, LDL-C and HDL-C: prevalence NR<br>Dyslipidemia: 23.3%
Bélanger [5]	Total Cholesterol, LDL-C, HDL-C, TG, dyslipidemia	High TG: 12%<br>High total Cholesterol: 10%<br>Low HDL-C: 16%<br>High LDL-C: 10%<br>Dyslipidemia: 30%
Bérard [6]	Total Cholesterol, LDL-C, HDL-C, TG, apolipoprotein A1 and B100, dyslipidemia	For total Cholesterol, LDL-C, HDL-C, TG, apolipoprotein A1 and B100: prevalence NR<br>Dyslipidemia: 41.1%
Bhatt [7]	Total Cholesterol, TG*	Hypertriglyceridemia in HSCT survivors vs. community controls: 45.4% vs. 1.2%, p <0.001<br>Hypertriglyceridemia conventional therapy vs. community controls: 20.9% vs. 18.1%, p = 0.057<br>Hypercholesterolemia in HSCT survivors vs. community controls: 46.9% vs. 30.8%, p = 0.001<br>Hypercholesterolemia conventional therapy vs. community controls: 23.9% vs. 30.8%, p = 0.69
Bis [8]	Total Cholesterol, LDL-C, HDL-C, TG	High total Cholesterol: 75%<br>High TG: 82.91%<br>High LDL-C: 37.6%<br>Low HDL-C: 62.96%
Bolier [9]	TG, HDL-C	High TG survivors vs. controls: 27.4% vs. 17.8%<br>Low HDL-C survivors vs. controls: 36.4% vs. 18%
Cacciotti [10]	Total Cholesterol, HDL-C, TG, dyslipidemia	High TG: 17%<br>Low HDL-C: 25%<br>High total Cholesterol: 75%<br>Dyslipidemia: 25%
Cepelova [11]	Total Cholesterol, LDL-C, HDL-C, TG, dyslipidemia	Low HDL-C survivors vs. controls: 17.5% vs. 13.3%<br>High TG survivors vs. controls: 25% vs. 13.3%

		High total Cholesterol, LDL-C: prevalence NR
Cooksey [13]	TG, HDL-C, non-HDL-C	High TG in irradiated vs. non-irradiated: 25% vs. 8.6%<br>Low HDL-C in irradiated vs. non-irradiated: 25% vs. 14.6%<br>Non-HDL: prevalence NR
Das [14]	HDL-C, LDL-C, VLDL-C, TG	Abnormal HDL-C, LDL-C, VLDL-C, TG: prevalence NR
Das [15]	HDL-C, TG	Low HDL-C: 40 %<br>High TG: 26 %
Delorme [16]	Total Cholesterol, LDL-C, HDL-C	High total Cholesterol intervention vs. control group: 11.9% vs. 11.7%, p = 1.0<br>High LDL-C control intervention vs. control group: 9.8 vs. 11.7, p = 0.773<br>Low HDL-C control intervention vs. control group: 19.5% vs. 19.5%, p = 1.0
England [17]	LDL-C, HDL-C, TG<br>Dyslipidemia	LDL-C, HDL-C, TG: prevalence NR<br>Dyslipidemia in total cohort: 41.8 %, adults 46.9 %, children 30.2 %
Faber [18]	LDL-C, HDL-C, TG<br>Dyslipidemia	LDL-C, HDL-C, TG: prevalence NR<br>Dyslipidemia : 28.3%
Felicetti [20]	Total Cholesterol, TG	High total Cholesterol: 20%<br>High TG: 6%
Goldberg [21]	LDL-C, HDL-C, non-HDL-C, TG	High LDL-C: 8.7%<br>High non-HDL-C: 11.5%<br>Low HDL-C: 60.5%<br>High TG: 17.2%
Hwang [23]	Total Cholesterol, LDL-C, HDL-C, TG, dyslipidemia	Total Cholesterol, LDL-C, HDL-C, TG: prevalence NR<br>Dyslipidemia in total cohort: 22%, prepubertal age at HSCT: 18%, post pubertal age at HSCT: 26%
Javalkar [24]	Total Cholesterol, LDL-C, HDL-C, TG	High LDL-C survivors vs. controls: 47.6% vs. 48.1%<br>High TG survivors vs. controls: 84.3% vs. 49.4%<br>Low HDL-C survivors vs. controls: 62.5% vs. 35.2%<br>High Cholesterol survivors vs. controls: 62.8% vs. 58%
Jin [25]	Total Cholesterol, LDL-C, HDL-C, TG	High total Cholesterol and LDL-C: prevalence NR<br>High TG survivors vs. controls: 26.8% vs. 16.8%<br>Low HDL-C survivors vs. controls: 25.4% vs. 20.8%
Lee [30]	LDL-C, HDL-C, TG, dyslipidemia	High LDL-C: 2.1%<br>High TG: 2.4%<br>Dyslipidemia: 3.2%
Levy [31]	LDL-C, HDL-C, TG, dyslipidemia	Total cohort:<br>- High LDL-C: 17.4%

		- High TG: 12.2%<br>- Low HDL-C: 23.1%<br>- Dyslipidemia: 41.3%<br>Adults:<br>- High LDL-C: 22.2%<br>- High TG: 13%<br>- Low HDL-C: 26.5%<br>- Dyslipidemia: 48.1%<br>Children:<br>- High LDL-C: 8.2%<br>- High TG: 10.6%<br>- Low HDL-C: 16.5%<br>- Dyslipidemia: 28.2%
Lubas [32]	Total Cholesterol, LDL-C, HDL-C, TG, dyslipidemia	Total Cholesterol, LDL-C, HDL-C, TG: prevalence NR<br>Dyslipidemia: 12.7%
Mogensen [34]	Total Cholesterol, LDL-C, HDL-C, TG, dyslipidemia	High TG: 58%<br>High total Cholesterol: 5%<br>Low LDL-C: 13%<br>Dyslipidemia: 99%
Mohapatra [35]	HDL-C, TG	High TG: 19.7%<br>Low HDL-C: 36.8%
Morel [36]	Total Cholesterol, LDL-C, HDL-C, TG, dyslipidemia	Low HDL-C: 23.1%<br>High TG: 12.2%<br>High LDL-C: 17.4%<br>High total Cholesterol: prevalence NR<br>Dyslipidemia: 41.4 %
Nirmal [39]	HDL-C, TG	Low HDL-C: 28.2%<br>High TG: 24.9%
Oudin [40]	HDL-C, TG	Low HDL-C: 36.4%<br>High TG: 29.6%
Özdemir [41]	Total Cholesterol, LDL-C, HDL-C, TG, dyslipidemia	Low HDL-C: 14%<br>High LDL-C: 6%<br>High TG: 4%<br>High total Cholesterol: 4%<br>Dyslipidemia: 38%

Persson [42]	TG	TG ≥5× UNL at least once: 65.3%
Pluimakers [43]	LDL-C, HDL-C, TG<br>Apolipoprotein(apo)-A1, apoB, Leptin, Adiponectin, Lipoprotein(a)	High TG survivors vs. controls: 23% vs. 10%<br>Low HDL-C survivors vs. controls: 29% vs. 18%<br>Apolipoprotein(apo)-A1, apoB, Leptin, Adiponectin, Lipoprotein(a) (Lp (a)): prevalence NR
Pranjic [44]	Total Cholesterol, LDL-C, HDL-C, TG	High total Cholesterol, LDL-C, TG: prevalence NR<br>Low HDL-C: 44.4%
Saultier [46]	HDL-C, TG	Low HDL-C: 26.8%<br>High TG: 11.7%<br>Combination of low HDL and high TG: 5.2%
Schindera [47]	HDL-C, TG	Low HDL-C: 18 %<br>High TG: 19 %
Schmidt [48]	TG	High TG: 7 % (overall), 4.31 % (<10 years), 12.2 % (>10 years), 6 % (>14 years)
Wei [51]	HDL-C, TG	High TG in chemotherapy group with HSCT/TBI: 48%<br>High TG in chemotherapy group without HSCT/ TBI: 10%<br>Low HDL in chemotherapy group with HSCT/TBI: 57%<br>Low HDL in chemotherapy group without HSCT/TBI: 27%
Zareifar [52]	HDL-C, TG	Low HDL-C (≤5th percentile): 45.28%<br>High TG (≥95th percentile): 32.07 %

Abbreviations: HDL-C, high-density lipoprotein; LDL-C, low-density lipoprotein; TG, triglyceride

Supplementary Table S5: Results from case reports and small case series reporting about interventions performed during treatment and at diagnosis of dyslipidemia

Author	Intervention	Results
Ciolli [12]	Intervention: L-carnitine 2 g twice a day intramuscularly for 5 days, fenofibrate 145 mg/day orally and omega-3 1000mg three times a day orally and low-fat diet. Start after detection of hypertriglyceridemia and hypercholesterinemia<br>Omega-3 continues as prophylaxis; unclear how long L-carnitine and fenofibrate were given	"Quick normalization" under intervention one. After one week, TG and total Cholesterol was back to normal levels
Fachin [19]	Intervention 1: Continuous insulin-dextrose infusion with reduced triglyceride levels<br>Intervention 2: Evinacumab started at 15 mg/kg every four weeks	Max. level: TG up to 6000 mg/dL, total Cholesterol up to 2900 mg/dL.<br>After insulin-dextrose infusion: triglyceride levels 1300 mg/dL in 1 week<br>After start of Evinacumab: reduction of TG to 154 mg/dL in 24 h. Continuation of Evinacumab, triglyceride levels remained below 350 mg/dL. After 8 months (still under Evinacumab) triglyceride levels of 167 mg/dL at the last follow-up
Heenan [22]	Interventions: Gemfibrozil, Atorvastatin and fish oil capsules	No trajectory of lipid values described
Kaplan [26]	Intervention: Fenofibrate (Lipanthyl® 267 mg capsules)	Total serum total Cholesterol:<br>- before starting AML treatment: 131.2 mg/dL<br>- after starting AML treatment but before fenofibrate: 727.6 mg/dL<br>- 10 days after start of fenofibrate: 265 mg/dL<br>Triglyceride levels:<br>- before starting AML treatment: 89.8 mg/dL<br>- after starting AML treatment but before fenofibrate: 6015.6 mg/dL<br>- 10 days after fenofibrate start: 743 mg/dL
Lau [28]	Intervention 1: Low fat diet; lipid omitted from the total parenteral nutrition (case 1)<br>Intervention 2: IV infusion of SMOFlipid (containing 3 % fish oil which is high in eicosapentaenoic acid [EPA] and docosahexaenoic acid [DHA]) at 0.5 g/kg/day (5 days) (case 1 and 2)	Case 1: Before SMOFlipid: Elevated serum triglyceride at 8.86 mmol/L; further increase despite conservative measures and patient became delirious (14.97 mmol/L). After SMOFlipid: triglyceride level reduced rapidly over the next 2 days<br>Case 2: Before SMOFlipid (day 28 induction chemotherapy): triglyceride measured retrospectively (1.77-3.07 mmol/L). Day 3 of admission: fasting TG elevated: 15.49 mmol/L. Two days after SMOFlipid: TG declined to 5.77 mmol/L
Mayerhofer [33]	Intervention: Lipid apheresis	Severe hypertriglyceridemia diagnosed at day 32 and apheresis started

		Improvement in TG levels: day 32: 7700mg/dL to 1200 mg/dL: day 34: TG remained < 500mg/dL
Nagayama [37]	Intervention: Metreleptin (0.04 mg/kg) daily for 1 week, then 0.08 mg/kg s.c. daily	-
Salvador [45]	Intervention 1: Low-fat diet<br>Intervention 2: Omega-3 and Acipimox for patients with TG >3000 mg/dL despite Intervention 1<br>Combination of omega-3 fatty acids: Omacor 1000 mg capsules, 1 capsule/d to a maximum dose of 3 capsules/d in 1 adolescent patient) Plus analogue of nicotinic acid (Acipimox, Olbetam 250 mg capsules, 2 capsules/d as maximum dose)<br>Intervention 3: Omega-3, Acipimox and Fibrate for patients with TG > 3000 mg/dL despite Intervention 1 and 2.<br>Intervention 2 plus a fibrate (Bezalip 200 mg tablets, 1 tablet/d)	Hypertriglyceridemia<br>- moderate: 500-1000 mg/dL (65.9%)<br>- severe: 1000-2500 mg/dL (19.5%)<br>- very severe: >2500 mg/dL (14.6%)
Zawitowska [53]	Intervention Case 1 + 2 First Line: Insulinotherapy (10 % glucose infusion), low-fat diet, and low molecular weight heparin subcutaneously.<br>Intervention Case 1 Second Line: Plasmapheresis applied with fresh frozen plasma as replacement fluid; continued for three days.<br>Intervention Case 2 Second Line: Plasmapheresis with 5 % albumin as replacement fluid; continued for three days	-

Supplementary Tables S6: Critical appraisal of studies included in final systematic review according to JBI (https://jbi.global/critical-appraisal-tools, accessed 27 August 2025)

Randomized Controlled Trials

First author	Year	Was true randomization used for assignment of participants to treatment groups?	Was allocation to treatment groups concealed?	Were treatment groups similar at the baseline?	Were participants blind to treatment assignment?	Were those delivering the treatment blind to treatment assignment?	Were treatment groups treated identically other than the intervention of interest?	Were outcome assessors blind to treatment assignment?	Were outcomes measured in the same way for treatment groups?	Were outcomes measured in a reliable way?	Was follow up complete and if not, were differences between groups in terms of their follow up adequately described and analyzed?	Were participants analyzed in the groups to which they were randomized?	Was appropriate statistical analysis used?	Was the trial design appropriate and any deviations from the standard RCT design (individual randomization, parallel groups) accounted for in the conduct and analysis of the trial?	Quality
Barbosa-Cortes [2]	2023	Yes	Unclear	Yes	Yes	Yes	Yes	Yes	Yes	Yes	Yes	Yes	No	Yes	2
Warris [50]	2016	Yes	Yes	NA	Yes	Yes	Yes	Unclear	Yes	Yes	Yes	Yes	Yes	Yes	2

Analytical Cross-Sectional Studies

First author	Year	Were the criteria for inclusion in the sample clearly defined?	Were the study subjects and the setting described in detail?	Was the exposure measured in a valid and reliable way?	Were objective, standard criteria used for measurement of the condition?	Were confounding factors identified?	Were strategies to deal with confounding factors stated?	Were the outcomes measured in a valid and reliable way?	Was appropriate statistical analysis used?	Quality
Armstrong [1]	2015	Yes	Yes	Yes	Yes	NA	NA	Yes	Yes	2
Barbosa-Cortes [3]	2017	Yes	Yes	Yes	Yes	Unclear	No	Yes	Yes	2
Bayram [4]	2017	Yes	Yes	Yes	Yes	No	No	Yes	Yes	2
Bolier [9]	2024	Yes	Yes	Yes	Yes	Yes	Yes	Yes	Yes	1
Cacciotti [10]	2020	Yes	Yes	Yes	Yes	No	No	Yes	Yes	2
Cepelova [11]	2019	Yes	Yes	Yes	Yes	Unclear	No	Yes	Yes	2
Cooksey [13]	2018	Yes	Yes	Yes	Yes	No	No	Yes	Yes	2
Das [14]	2024	Yes	Unclear	Yes	Yes	Yes	No	Yes	No	3
Das [15]	2024	Yes	Unclear	Yes	Yes	Yes	No	Yes	No	3
England [17]	2017	Yes	Yes	Yes	Yes	NA	NA	Yes	Yes	2
Faber [18]	2017	Yes	Yes	Yes	Yes	No	No	Yes	Yes	2
Levy [31]	2017	Yes	Yes	Yes	Yes	Yes	Yes	Yes	Yes	1
Lubas [32]	2021	Yes	Yes	Yes	Yes	Yes	Yes	Yes	Yes	1
Mohapatra [35]	2026	Yes	Yes	Yes	Yes	No	No	Yes	No	3
Morel [36]	2019	Yes	Yes	Yes	Yes	Yes	Yes	Yes	Yes	1

Oudin [40]	2025	Yes	Yes	Yes	Yes	No	No	Yes	Yes	2
Pluimakers [43]	2020	Yes	Yes	Yes	Yes	Yes	Yes	Yes	Yes	1
Pranjic [44]	2025	Yes	Yes	Yes	Yes	No	No	Yes	Yes	2
Salvador [45]	2018	Yes	Yes	Yes	Yes	No	No	Yes	Yes	2
Saultier [46]	2021	Yes	Yes	Yes	Yes	Yes	Yes	Yes	Yes	1
Schindera [47]	2021	Yes	Yes	Yes	Yes	NA	NA	Yes	Yes	2
Wei [51]	2016	Yes	Yes	Yes	Yes	No	No	Yes	Yes	2

Case Control Studies

First author	Year	Were the groups comparable other than the presence of disease in cases or the absence of disease in controls?	Were cases and controls matched appropriately?	Were the same criteria used for identification of cases and controls?	Was exposure measured in a standard, valid and reliable way?	Was exposure measured in the same way for cases and controls?	Were confounding factors identified?	Were strategies to deal with confounding factors stated?	Were outcomes assessed in a standard, valid and reliable way for cases and controls?	Was the exposure period of interest long enough to be meaningful?	Was appropriate statistical analysis used?	Quality
Bélanger [5]	2021	Yes	Yes	NA	Yes	NA	Unclear	No	Yes	Yes	No	3
Bhatt [7]	2021	Unclear	No	NA	Yes	NA	Yes	Yes	Unclear	Yes	Yes	3
Laumann [29]	2020	Yes	Yes	No	Yes	Yes	No	No	Unclear	Yes	Unclear	3
Özdemir [41]	2018	Yes	Unclear	NA	Yes	NA	No	No	Yes	Yes	Yes	3

Cohort Studies

First author	Year	Were the two groups similar and recruited from the same population?	Were the exposures measured similarly to assign people to both exposed and unexposed groups?	Was the exposure measured in a valid and reliable way?	Were confounding factors identified?	Were strategies to deal with confounding factors stated?	Were the groups/participants free of the outcome at the start of the study (or at the moment of exposure)?	Were the outcomes measured in a valid and reliable way?	Was the follow up time reported and sufficient to be long enough for outcomes to occur?	Was follow up complete, and if not, were the reasons to loss to follow up described and explored?	Were strategies to address incomplete follow up utilized?	Was appropriate statistical analysis used?	Quality
Bélanger [5]	2021	Yes	Yes	Yes	Unclear	No	Unclear	Yes	Yes	Yes	NA	No	3
Bérard [6]	2020	NA	NA	Yes	Yes	Yes	Unclear	Yes	Yes	Yes	NA	Yes	3
Bis [8]	2020	NA	NA	Yes	No	No	Unclear	Yes	Yes	Yes	NA	Yes	3
Delorme [16]	2024	Yes	Yes	Yes	No	No	Unclear	Yes	Unclear	Yes	NA	Yes	3
Faber [18]	2017	No	NA	Yes	No	No	No	Yes	Yes	Yes	Yes	Yes	3
Felicetti [20]	2015	NA	NA	Yes	Yes	Yes	Unclear	Yes	Yes	Yes	Yes	Yes	3
Goldberg [21]	2023	NA	Yes	Yes	Yes	Yes	Yes	Yes	Yes	Yes	Yes	Yes	2
Hwang [23]	2023	Yes	Yes	Yes	Yes	Yes	Unclear	Yes	Yes	Yes	NA	Yes	2
Javalkar [24]	2022	NA	Yes	Yes	Yes	Yes	No	Yes	Unclear	Yes	Yes	Yes	3
Jin [25]	2023	NA	Yes	Yes	Yes	Yes	Unclear	Yes	Yes	Yes	Yes	Yes	2
Lee [30]	2021	NA	NA	Yes	No	No	Unclear	Yes	Yes	Yes	NA	Yes	3
Levy [31]	2017	Yes	Yes	Yes	Yes	Yes	Unclear	Yes	Yes	Yes	NA	Yes	2
Lubas [32]	2021	Yes	Yes	Yes	Yes	Yes	Unclear	Yes	Yes	Yes	NA	Yes	2
Napartuk [38]	2023	NA	NA	Yes	Unclear	No	Unclear	Yes	Yes	Yes	NA	Yes	3
Schmidt [48]	2021	Yes	Yes	Yes	Unclear	Yes	Unclear	Unclear	Yes	Yes	NA	Yes	3
Zareifar [52]	2017	NA	NA	Yes	NA	NA	Unclear	Yes	Yes	Yes	NA	NA	3

Case Reports

First author	Year	Were patient's demographic characteristics clearly described?	Was the patient's history clearly described and presented as a timeline?	Was the current clinical condition of the patient on presentation clearly described?	Were diagnostic tests or assessment methods and the results clearly described?	Was the intervention(s) or treatment procedure(s) clearly described?	Was the post-intervention clinical condition clearly described?	Were adverse events (harms) or unanticipated events identified and described?	Does the case report provide takeaway lessons?	Quality
Ciolli [12]	2024	Yes	Yes	Yes	Yes	Yes	Yes	No	Unclear	2
Fachin [19]	2023	Yes	Yes	Yes	Yes	Yes	Yes	Yes	Yes	1
Heenan [22]	2021	No	Yes	Yes	Unclear	Yes	No	No	Unclear	3
Kaplan [26]	2024	No	Yes	Yes	Yes	Yes	Yes	No	Unclear	3
Khera [27]	2022	Yes	Yes	Yes	Yes	Yes	Yes	NA	Unclear	2
Lau [28]	2021	Yes	Yes	Yes	Yes	Yes	Yes	No	Unclear	2
Mayerhofer [33]	2020	No	Yes	Yes	Yes	Yes	No	Yes	Yes	2
Nagayama [37]	2019	No	Yes	Yes	Yes	Yes	Unclear	No	Yes	3
Sonowal [49]	2019	Yes	Yes	Yes	Unclear	Yes	Yes	No	Unclear	3
Zawitowska [53]	2019	Yes	Yes	Yes	Yes	Yes	Yes	No	Unclear	2

Studies reporting prevalence data

First Author	Year	Was the sample frame appropriate to address the target population?	Were study participants sampled in an appropriate way?	Was the sample size adequate?	Were the study subjects and the setting described in detail?	Was the data analysis conducted with sufficient coverage of the identified sample?	Were valid methods used for the identification of the condition?	Was the condition measured in a standard, reliable way for all participants? and described?	Was there appropriate statistical analysis?	Was the response rate adequate, and if not, was the low response rate managed appropriately?	Quality
Mogensen [34]	2024	Yes	Yes	Yes	Yes	Yes	Yes	Yes	Yes	Yes	1
Nirmal [39]	2021	Yes	Yes	Yes	Yes	Yes	Yes	Yes	Yes	Yes	1
Persson [42]	2017	Yes	Yes	Unclear	Yes	Yes	Yes	Yes	Yes	Yes	2

References

[1] Armstrong GT, Joshi VM, Ness KK, Marwick TH, Zhang N, Srivastava D, et al. Comprehensive Echocardiographic Detection of Treatment-Related Cardiac Dysfunction in Adult Survivors of Childhood Cancer. J Am Coll Cardiol 2015;65:2511–22. https://doi.org/10.1016/j.jacc.2015.04.013.

[2] Barbosa-Cortes L, Atilano-Miguel S, Martin-Trejo JA, Jiménez-Aguayo E, Martínez-Becerril FI, López-Alarcón M, et al. Effect of long-chain omega-3 polyunsaturated fatty acids on cardiometabolic factors in children with acute lymphoblastic leukemia undergoing treatment: a secondary analysis of a randomized controlled trial. Front Endocrinol 2023;14:1120364. https://doi.org/10.3389/fendo.2023.1120364.

[3] Barbosa-Cortés L, López-Alarcón M, Mejía-Aranguré JM, Klünder-Klünder M, Del Carmen Rodríguez-Zepeda M, Rivera-Márquez H, et al. Adipokines, insulin resistance, and adiposity as a predictors of metabolic syndrome in child survivors of lymphoma and acute lymphoblastic leukemia of a developing country. BMC Cancer 2017;17:125. https://doi.org/10.1186/s12885-017-3097-8.

[4] Bayram C, Yaralı N, Fettah A, Demirel F, Tavil B, Kara A, et al. Evaluation of Endocrine Late Complications in Childhood Acute Lymphoblastic Leukemia Survivors: A Report of a Single-Center Experience and Review of the Literature. Turk J Hematol 2017;34:40–5. https://doi.org/10.4274/tjh.2015.0332.

[5] Bélanger V, Morel S, Napartuk M, Bouchard I, Meloche C, Curnier D, et al. Abnormal HDL lipid and protein composition following pediatric cancer treatment: an associative study. Lipids Health Dis 2023;22:72. https://doi.org/10.1186/s12944-023-01822-2.

[6] Bérard S, Morel S, Teasdale E, Shivappa N, Hebert JR, Laverdière C, et al. Diet Quality Is Associated with Cardiometabolic Outcomes in Survivors of Childhood Leukemia. Nutrients 2020;12:2137. https://doi.org/10.3390/nu12072137.

[7] Bhatt NS, Baassiri MJ, Liu W, Bhakta N, Chemaitilly W, Ehrhardt MJ, et al. Late outcomes in survivors of childhood acute myeloid leukemia: a report from the St. Jude Lifetime Cohort Study. Leukemia 2021;35:2258–73. https://doi.org/10.1038/s41375-021-01134-3.

[8] Bis G, Szlasa W, Sondaj K, Zendran I, Mielcarek-Siedziuk M, Barg E. Lipid Complications after Hematopoietic Stem Cell Transplantation (HSCT) in Pediatric Patients. Nutrients 2020;12:2500. https://doi.org/10.3390/nu12092500.

[9] Bolier, M.; Pluimakers , V.G.; De Winter, D.T.C.; Fiocco, M.; Van Den Berg, S.A.A.; Bresters, D.; van Dulmen-den Broeder, E.; van der Heiden-van der Loo, M.; Höfer, I.; Janssens, G.O.; et al. Prevalence and determinants of dyslipidemia in 2338 Dutch childhood cancer survivors: A DCCS-LATER 2 study. Eur. J. Endocrinol. 2024, 191, 588–603. https://doi.org/10.1093/ejendo/lvae149.

[10] Cacciotti C, Ali M, Bartels U, Wasserman JD, Kapllani E, Krueger J, et al. Early signs of metabolic syndrome in pediatric central nervous system tumor survivors after high-dose chemotherapy and autologous stem-cell transplantation and radiation. Childs Nerv Syst 2021;37:1087–94. https://doi.org/10.1007/s00381-020-04971-2.

[11] Cepelova M, Kruseova J, Luks A, Capek V, Cepela P, Potockova J, et al. Accelerated atherosclerosis, hyperlipoproteinemia and insulin resistance in long-term survivors of Hodgkin lymphoma during childhood and adolescence. Neoplasma 2019;66:978–87. https://doi.org/10.4149/neo_2019_190115N45.

[12] Ciolli G, Pasquini A, Mannelli F, Scappini B, Gianfaldoni G, Quinti E, et al. Successful rechallenge with Erwinia chrysanthemi asparaginase after pegaspargase-induced hypertriglyceridemia: a case report. Ther Adv Hematol 2024;15:20406207241270846. https://doi.org/10.1177/20406207241270846.

[13] Cooksey R, Wu SY, Klesse L, Oden JD, Bland RE, Hodges JC, et al. Metabolic syndrome is a sequela of radiation exposure in hypothalamic obesity among survivors of childhood brain tumors. J Investig Med 2019;67:295–302. https://doi.org/10.1136/jim-2018-000911.

[14] Das G, Setlur K, Jana M, Ramakrishnan L, Jain V, Meena JP, et al. Serum Adipokines as Biomarkers for Surveillance of Metabolic Syndrome in Childhood Acute Lymphoblastic Leukemia Survivors in Low Middle-Income Countries. Nutr Cancer 2024;76:262–70. https://doi.org/10.1080/01635581.2023.2301139.

[15] Das G, Setlur K, Jana M, Ramakrishnan L, Jain V, Meena JP, et al. Sarcopenic obesity in survivors of childhood acute lymphoblastic leukemia: prevalence, risk factors, and implications for cancer survivors. Support Care Cancer 2024;32:826. https://doi.org/10.1007/s00520-024-09025-w.

[16] Delorme J, Dima A, Bélanger V, Napartuk M, Bouchard I, Meloche C, et al. Impact of Early Nutritional Intervention During Cancer Treatment on Dietary Intakes and Cardiometabolic Health in Children and Adolescents. Cancers 2025;17:157. https://doi.org/10.3390/cancers17010157.

[17] England J, Drouin S, Beaulieu P, St-Onge P, Krajinovic M, Laverdière C, et al. Genomic determinants of long-term cardiometabolic complications in childhood acute lymphoblastic leukemia survivors. BMC Cancer 2017;17:751. https://doi.org/10.1186/s12885-017-3722-6.

[18] Faber J, Wingerter A, Neu MA, Henninger N, Eckerle S, Münzel T, et al. Burden of cardiovascular risk factors and cardiovascular disease in childhood cancer survivors: data from the German CVSS-study. Eur Heart J 2018;39:1555–62. https://doi.org/10.1093/eurheartj/ehy026.

[19] Fachin A, De Carlo C, Maestro A, Zanon D, Barbi E, Maximova N. Rapid Resolution of Life-Threatening Hypertriglyceridemia after Evinacumab Administration in a Pediatric HSCT Recipient: A Case Report. Pharmaceuticals 2023;16:1069. https://doi.org/10.3390/ph16081069.

[20] Felicetti F, D'Ascenzo F, Moretti C, Corrias A, Omedè P, Marra WG, et al. Prevalence of cardiovascular risk factors in long-term survivors of childhood cancer: 16 years follow up from a prospective registry. Eur J Prev Cardiol 2015;22:762–70. https://doi.org/10.1177/2047487314529348.

[21] Goldberg JF, Hyun G, Ness KK, Dixon SB, Towbin JA, Rhea IB, et al. Dyslipidemia and cardiovascular disease among childhood cancer survivors: a St. Jude Lifetime Cohort report. JNCI J Natl Cancer Inst 2024;116:408–20. https://doi.org/10.1093/jnci/djad222.

[22] Heenan JM, Hooper AJ, Burnett JR, Cooney J. l-asparaginase-induced biochemical toxicities in young adults with acute lymphoblastic leukaemia and T-lymphoblastic lymphoma. Pathology (Phila) 2021;53:924–6. https://doi.org/10.1016/j.pathol.2021.02.015.

[23] Hwang S, Lee Y, Yoon J-H, Kim JH, Kim H, Koh K-N, et al. Long-term endocrine sequelae after hematopoietic stem cell transplantation in children and adolescents. Ann Pediatr Endocrinol Metab 2024;29:109–18. https://doi.org/10.6065/apem.2346046.023.

[24] Javalkar K, Huang Y, Lyon SM, Palfrey H, Hartz J, Chen MH, et al. Clinical response to lifestyle counseling for dyslipidemia and elevated blood pressure in childhood cancer survivors. Pediatr Blood Cancer 2023;70:e30034. https://doi.org/10.1002/pbc.30034.

[25] Jin HY, Lee JA, Park M, Park HJ. Prevalence and Risk Factors of Metabolic Syndrome Components in Childhood Cancer Survivors. J Adolesc Young Adult Oncol 2023;12:224–31. https://doi.org/10.1089/jayao.2021.0227.

[26] Kaplan Koruk RH, Kocabora MS, Erdur SK, Yaman Y. Lipemia retinalis following FLAG-Ida protocol in an 11-year-old patient with acute myeloid leukemia. Eur J Ophthalmol 2025;35:NP5–9. https://doi.org/10.1177/11206721241287347.

[27] Khera S, Kapoor R, Sunder S, Mahajan D. Grade 4 very severe hypertriglyceridaemia at diagnosis in a child with acute lymphoblastic leukaemia. BMJ Case Rep 2022;15:e245820. https://doi.org/10.1136/bcr-2021-245820.

[28] Lau SCD, Loh C-K, Alias H. Case Report: The Use of Intravenous SMOFlipid Infusion to Treat Severe Asparaginase-Induced Hypertriglyceridemia in Two Pediatric Acute Lymphoblastic Leukemia Patients. Front Pediatr 2021;9:660627. https://doi.org/10.3389/fped.2021.660627.

[29] Laumann, R.D.; Iversen, T.; Mogensen, P.R.; Lauritzen, L.; Mølgaard, C.; Frandsen, T.L. Effect of Fish Oil Supplementation on Hyperlipidemia during Childhood Acute Lymphoblastic Leukemia Treatment—A Pilot Study. Nutr. Cancer 2021, 73, 1816–1820. https://doi.org/10.1080/01635581.2020.1803934.
[30] Lee Y, Shin J, Choi Y, Kim H, Koh K-N, Im HJ, et al. Endocrine Complications in Children and Adolescents With Non-Central Nervous System Solid Tumors. Front Endocrinol 2021;12:610730. https://doi.org/10.3389/fendo.2021.610730.
[31] Levy E, Samoilenko M, Morel S, England J, Amre D, Bertout L, et al. Cardiometabolic Risk Factors in Childhood, Adolescent and Young Adult Survivors of Acute Lymphoblastic Leukemia – A Petale Cohort. Sci Rep 2017;7:17684. https://doi.org/10.1038/s41598-017-17716-0.
[32] Lubas MM, Wang M, Jefferies JL, Ness KK, Ehrhardt MJ, Krull KR, et al. The Contribution of Stress and Distress to Cardiovascular Health in Adult Survivors of Childhood Cancer. Cancer Epidemiol Biomarkers Prev 2021;30:286–94. https://doi.org/10.1158/1055-9965.EPI-20-1183.
[33] Mayerhofer C, Speckmann C, Kapp F, Teufel-Schäfer U, Kluwe W, Schneider J, et al. Lipid Apheresis to Manage Severe Hypertriglyceridemia during Induction Therapy in a Child with Acute Lymphoblastic Leukemia. Pediatr Hematol Oncol 2020;37:530–8. https://doi.org/10.1080/08880018.2020.1756999.
[34] Mogensen PR, Grell K, Schmiegelow K, Overgaard UM, Wolthers BO, Mogensen SS, et al. Dyslipidemia at diagnosis of childhood acute lymphoblastic leukemia. PLOS ONE 2020;15:e0231209. https://doi.org/10.1371/journal.pone.0231209.
[35] Mohapatra S, Bansal D, Bhalla AK, Verma Attri S, Sachdeva N, Trehan A, et al. Is there an increased risk of metabolic syndrome among childhood acute lymphoblastic leukemia survivors? A developing country experience. Pediatr Hematol Oncol 2016;33:136–49. https://doi.org/10.3109/08880018.2016.1152335.
[36] Morel S, Amre D, Teasdale E, Caru M, Laverdière C, Krajinovic M, et al. Dietary Intakes Are Associated with HDL-Cholesterol in Survivors of Childhood Acute Lymphoblastic Leukaemia. Nutrients 2019;11:2977. https://doi.org/10.3390/nu11122977.
[37] Nagayama A, Ashida K, Moritaka K, Hidaka M, Gobaru M, Tanaka S, et al. Metreleptin Supplementation for Improving Lipid and Glycemic Profiles in Acquired Diabetes Lipodystrophy: A Case Report. J Endocr Soc 2019;3:2179–83. https://doi.org/10.1210/js.2019-00251.
[38] Napartuk M, Bélanger V, Bouchard I, Meloche C, Curnier D, Sultan S, et al. Improvement of Diet after an Early Nutritional Intervention in Pediatric Oncology. Children 2023;10:667. https://doi.org/10.3390/children10040667.
[39] Nirmal G, Thankamony P, Chellapam Sojamani G, Nair M, Rajeswari B, Varikkattu Rajendran P, et al. Prevalence and Risk Factors for Metabolic Syndrome Among Childhood Acute Lymphoblastic Leukemia Survivors: Experience From South India. J Pediatr Hematol Oncol 2021;43:e154–8. https://doi.org/10.1097/MPH.0000000000001856.
[40] Oudin C, Auquier P, Bertrand Y, Contet A, Kanold J, Sirvent N, et al. Metabolic syndrome in adults who received hematopoietic stem cell transplantation for acute childhood leukemia: an LEA study. Bone Marrow Transplant 2015;50:1438–44. https://doi.org/10.1038/bmt.2015.167.
[41] Özdemir ZC, Düzenli Kar Y, Demiral M, Sırmagül B, Bör Ö, Kırel B. The Frequency of Metabolic Syndrome and Serum Osteopontin Levels in Survivors of Childhood Acute Lymphoblastic Leukemia. J Adolesc Young Adult Oncol 2018;7:480–7. https://doi.org/10.1089/jayao.2017.0129.
[42] Persson L, Harila-Saari A, Hed Myrberg I, Heyman M, Nilsson A, Ranta S. Hypertriglyceridemia during asparaginase treatment in children with acute lymphoblastic leukemia correlates with antithrombin activity in adolescents. Pediatr Blood Cancer 2017;64:e26559. https://doi.org/10.1002/pbc.26559.

[43] Pluimakers VG, Van Waas M, Looman CWN, De Maat MP, De Jonge R, Delhanty P, et al. Metabolic syndrome detection with biomarkers in childhood cancer survivors. Endocr Connect 2020;9:676–86. https://doi.org/10.1530/EC-20-0144.

[44] Pranjić I, Sila S, Lulić Kujundžić S, Dodig M, Vestergaard Larsen A, Kranjčec I. Metabolic Sequelae and Quality of Life in Early Post-Treatment Period in Adolescents with Hodgkin Lymphoma. J Clin Med 2025;14:375. https://doi.org/10.3390/jcm14020375.

[45] Salvador C, Entenmann A, Salvador R, Niederwanger A, Crazzolara R, Kropshofer G. Combination therapy of omega-3 fatty acids and acipimox for children with hypertriglyceridemia and acute lymphoblastic leukemia. J Clin Lipidol 2018;12:1260–6. https://doi.org/10.1016/j.jacl.2018.05.021.

[46] Saultier P, Auquier P, Bertrand Y, Vercasson C, Oudin C, Contet A, et al. Metabolic syndrome in long-term survivors of childhood acute leukemia treated without hematopoietic stem cell transplantation: an L.E.A. study. Haematologica 2016;101:1603–10. https://doi.org/10.3324/haematol.2016.148908.

[47] Schindera C, Zürcher SJ, Jung R, Boehringer S, Balder JW, Rueegg CS, et al. Physical fitness and modifiable cardiovascular disease risk factors in survivors of childhood cancer: A report from the SURfit study. Cancer 2021;127:1690–8. https://doi.org/10.1002/cncr.33351.

[48] Schmidt M-P, Ivanov A-V, Coriu D, Miron I-C. L-Asparaginase Toxicity in the Treatment of Children and Adolescents with Acute Lymphoblastic Leukemia. J Clin Med 2021;10:4419. https://doi.org/10.3390/jcm10194419.

[49] Sonowal R, Gupta V. Severe hyperlipidemia in a case of acute lymphoblastic leukemia. Indian J Cancer 2019;56:180. https://doi.org/10.4103/ijc.IJC_724_18.

[50] Warris LT, Van Den Akker ELT, Bierings MB, Van Den Bos C, Zwaan CM, Sassen SDT, et al. Acute Activation of Metabolic Syndrome Components in Pediatric Acute Lymphoblastic Leukemia Patients Treated with Dexamethasone. PLOS ONE 2016;11:e0158225. https://doi.org/10.1371/journal.pone.0158225.

[51] Wei C, Hunt L, Cox R, Bradley K, Elson R, Shield J, et al. Identifying Cardiovascular Risk in Survivors of Childhood Leukaemia Treated with Haematopoietic Stem Cell Transplantation and Total Body Irradiation. Horm Res Paediatr 2017;87:116–22. https://doi.org/10.1159/000455046.

[52] Zareifar S, Haghpanah S, Shorafa E, Shakibazad N, Karamizadeh Z. Evaluation of Metabolic Syndrome and Related Factors in Children Affected by Acute Lymphoblastic Leukemia. Indian J Med Paediatr Oncol Off J Indian Soc Med Paediatr Oncol 2017;38:97–102. https://doi.org/10.4103/ijmpo.ijmpo_69_16.

[53] Zawitkowska J, Lejman M, Zaucha-Prażmo A, Sekuła N, Greczkowska-Chmiel T, Drabko K. Severe drug-induced hypertriglyceridemia treated with plasmapheresis in children with acute lymphoblastic leukemia. Transfus Apher Sci 2019;58:634–7. https://doi.org/10.1016/j.transci.2019.08.025.
